# Magnetic microscopy for operando imaging of battery dynamics

**DOI:** 10.1038/s41467-025-63409-y

**Published:** 2025-09-17

**Authors:** Stefan Pollok, Mohamad Khoshkalam, Fardin Ghaffari-Tabrizi, Fran Kurnia, Danni Wang, Siqi Li, Dominik B. Bucher, Jennifer L. M. Rupp, Dennis V. Christensen

**Affiliations:** 1 Department of Energy Conversion and Storage, Technical University of Denmark, Kongens Lyngby, Denmark; 2 Department of Chemistry, School of Natural Sciences, Technical University of Munich, Garching, Germany; 3 TUMint.Energy Research GmbH, Garching, Germany; 4 Institute for Advanced Study, Technical University of Munich, Garching, Germany

**Keywords:** Batteries, Materials chemistry, Quantum metrology, Imaging techniques

## Abstract

Battery development pivots around understanding the complex processes governing battery operation and degradation. Most degradation pathways link structural and chemical inhomogeneities with strongly heterogeneous carrier transport at the nano- and microscale, which remains challenging to resolve with current operando imaging techniques. Here, we provide a data-driven perspective on using operando magnetic microscopy to examine the charge and discharge cycles in lithium and post-lithium batteries. Through quantitatively imaging ionic and electronic current distributions and probing the associated chemical reactions at the nanoscale, valuable insights into battery inhomogeneities and degradations can be gained. The approach facilitates spatially resolving heterogeneous redox reactions, buried current distributions, and mechanistic contributions to short-circuit endurance in batteries.

## Abstract (web summary)

Quantum sensing of lithium and post-lithium batteries is introduced to visualize how electrons and ions flow and react during operation. Here, authors show how this facilitates operando imaging of heterogeneous redox reactions, buried current distributions, and dendrite formation.

## Introduction

Batteries play an increasing role in the green transition by enabling the electrification of transportation and balancing sustainable energy production with demands. However, the global pursuit of high-performing batteries faces immense difficulties in achieving rapid charging, high energy densities, low cost, excellent lifetime, and safe operation simultaneously. This has fueled active research in emerging battery architectures and chemistries as well as a rapid surge in optimizing prevalent lithium-ion batteries^1,2^. Universal to all battery architectures is a general need for homogeneous battery operation, which limits degradation by diminishing the difference between overactive and inactive regions^3^. In practice, however, batteries are notoriously heterogeneous in their operation^4–7^, which remains one of the largest bottlenecks in understanding the local processes and perfecting their operation. The knowledge gap results from the buried and air-sensitive nature of batteries, which blocks direct access to understanding the local processes unfolding at the nanoscale. Particularly, solid-state and hybrid batteries offer high-performing prospects as next-generation batteries, but their operation is often determined by interface processes that are challenging to image as they are buried between solids with characteristic feature lengths on the nanoscale^8,9^.

Despite structural and chemical operando techniques with sub-surface imaging capabilities being routinely used to gain nanoscale insight into the battery operation^10–12^, there is a general lack of non-invasive tools for visualizing buried functional processes in action at the nanoscale. In particular, we lack tools for visualizing the buried flow of electrons and ions in batteries and their solid-state components, let alone correlative tools to understand the complex interplay between heterogeneous chemical reactions and heterogeneous charge transport.

Here, we propose to use nano- and microscale magnetic imaging as a functional tool for visualizing the buried processes in batteries of varying architectures and chemistries. Supported by numerical simulations, we demonstrate the prospects of magnetic imaging for visualizing how active ions and electrons flow and chemically react at the nanoscale during the full battery charge/discharge cycle. The insights gained from the magnetic properties and carrier characteristics are discussed for battery electrodes and electrolytes, which form a blueprint for magnetic imaging of iono-electronic charge carrier dynamics.

## Inhomogeneous battery operation and degradation

**Fig. 1:**
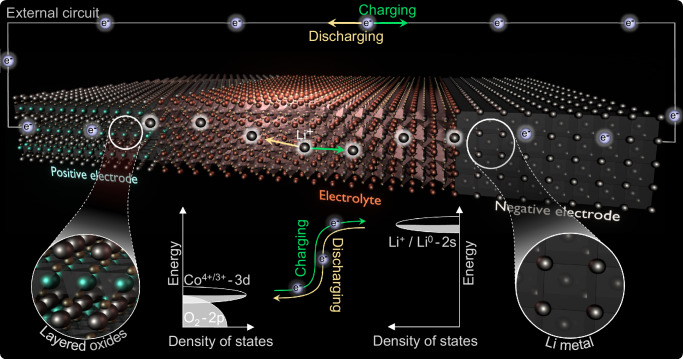
Battery operation. Batteries store and release energy by moving the active ions and electrons between a low-energy position in the positive electrode and a high-energy position in the negative electrode. The ions are transferred across the electrolyte, whereas electrons take up or release energy from an external circuit. This promotes or demotes electrons from low-energy 3d states in the transition metals of the positive electrode such as Co in the layered oxide LiCoO_2_ to high-energy states in the negative electrode related to the reduction of lithium ions to lithium metal.

Batteries store and release energy by moving mobile ions and electrons between a low-energy position in the positive electrode and a high-energy position in the negative electrode. Ions move between the electrodes solely by moving across an electrolyte, whereas electrons move in an external circuit to perform useful work. Conventional lithium-ion batteries typically comprise a liquid electrolyte paired with stable graphite at the negative electrode and either cost-effective LiFePO_4_ or an energy-dense layered oxide at the positive electrode, such as LiCoO_2_ and Ni-rich LiNi_x_Mn_y_Co_1-x-y_O_2_^1,2^. In Fig. 1, we schematically depict the conceptually similar charging/discharging process in solid-state batteries comprising the layered oxide LiCoO_2_ at the positive electrode and a lithium metal at the negative electrode. The use of solid electrolytes such as Li_7_La_3_Zr_2_O_12_ (LLZO) increases the chemical stability toward highly reactive lithium metal, which unlocks the high energy densities of metal negative electrodes^13,14^. During charging, electrons are removed from the 3d state of Co in LiCoO_2_ and transferred through the external circuit to the negative electrode. Lithium ions are simultaneously transferred from the 2D lithium planes within LiCoO_2_ to the negative electrode through the electrolyte. Lithium ions and electrons unite at the negative electrode to form lithium metal.

The ideal battery has a homogeneous operation characterized by homogeneous charge transport and homogeneous chemical reactions from the nano- to macroscale. This eliminates capacity loss from inactive regions while limiting degradation from overactive regions where, e.g., irreversible degradation occurs when cycling Li_x_CoO_2_ below x < 0.5^15–17^. Strong inhomogeneities, however, typically govern the operation of batteries and arguably every key degradation mechanism. Such inhomogeneities are often self-enhancing as heterogeneous charge transport in the battery promotes spatially varying chemical reaction rates, which, in turn, may change the local ionic and electronic conductivity to further amplify the transport heterogeneity^5,7,18^. This emphasizes the importance of local imaging of the electrochemical processes to gain the insight needed for mitigating degradation pathways and optimizing charging performance^5,7^.

The key spatial inhomogeneities associated with battery reactions at the negative electrode frequently lead to performance loss in batteries. Capacity degradation occurs when the active ions react to form metal inclusions in the electrolyte or solid-electrolyte interphases at the negative electrode|electrolyte interface^19,20^. The metal inclusions can grow to metal dendrites penetrating the electrolyte to eventually short-circuit the battery when reaching the positive electrode. The interdependence between the heterogeneous metal plating and heterogeneous charge transport causes a detrimental downward spiral. Here, locally enhanced electronic currents promote the conversion of active ions to metal at the tip of the metal dendrite, which, in turn, expands the region of enhanced electronic current to fuel even further metal growth until the battery short-circuits.

Spatial inhomogeneities also govern the electrolyte processes, particularly in solid-state batteries owing to the presence of grain boundaries with often widely different conducting and mechanical properties compared to the interior of the grains^21,22^. The inhomogeneous material properties typically determine the overall ionic conductivity and electronic leakage while also ultimately causing dendrites to grow along the grain boundary network^21,23^.

Lastly, the operation and degradation of positive electrodes also possess very heterogeneous characteristics arising particularly from redox heterogeneity^7,24^, spatially uneven strain evolutions^25^, cracking and dissolution of the positive electrode active material^25^, local phase transformation^15,26^, as well as the heterogeneous nature of the positive electrode active particles mixed with carbon additives and electrolyte^27,28^.

## Imaging inhomogeneities

Conventional characterization techniques such as electrochemical impedance spectroscopy, cyclic voltammetry, post-mortem analysis, and lab-scale X-ray diffraction lack the temporospatial capabilities to image internal processes in action at the nanoscale. Operando magnetic measurements have been successfully applied to non-invasively study the chemical redox processes, particularly in the magnetically active positive electrode active materials. However, these efforts are constrained to the macroscale either by conventional magnetic measurement on the entire sample or by coarse magnetic imaging on the macroscale^29–36^. Magnetic resonance imaging based on particularly nuclear magnetic resonance (NMR) has recently attracted considerable attention as a powerful tool for extracting versatile information about NMR-active lithium, yielding abundant information ranging from detailed descriptions of lithium diffusion to macroscale imaging of full batteries and lithium metal inclusion growth^37–39^. However, the spatial resolution in magnetic resonance imaging is preventing the nanoscale imaging of batteries, and although nanoscale NMR is being developed^40–43^, its use for battery characterization remains elusive.

Studies with excellent spatial resolution have been conducted using light-, electron-, and scanning probe-based tools. X-ray-based methods such as scanning transmission X-ray microscopy (STXM) and nanoscale X-ray tomography have yielded spectacular knowledge of, e.g., heterogeneous redox reactivity between and within individual positive electrode particles^6,24,44^ as well as reconstruction of the 3D morphology of solid-state batteries^45^ and metal inclusions^46^. The widespread adoption of these advanced X-ray techniques is, however, limited by the general need for synchrotron radiation, the beam invasiveness, and the weak scattering propensity of the light lithium metal^4,47^. As a result, optical interferometric scattering microscopy has emerged as a lab-scale nanoprobe of reactivity at the positive electrode^48^.

Techniques based on electron microscopy are powerful analytic tools for battery characterization owing to their exceptionally high spatial resolution. This suite of techniques has been used to, e.g., study mechanical interactions across interfaces^49^, chemical/structural changes^50–52^, and operando dendrite formation^5,49,53^. However, the beam-sensitivity and the shallow penetration depth of electrons pose strong limitations on these techniques^4^. A large variety of scanning probes have also been employed to study various aspects of batteries, ranging from leveraging strain response to detecting lithium reactivity at the electrodes using electrochemical strain microscopy^54,55^ and studying heterogeneous redox reactivity on the single particle level using scanning electrochemical microscopy^56^. Although very powerful, the scanning probe techniques typically remain insensitive to processes buried well within a material and many variants require exposed surfaces that are conducting or electrochemically active.

Importantly, state-of-the-art imaging techniques based on electrons, light, or scanning probes are generally not able to visualize the buried flow of both electrons and ions at the local scale, effectively constituting a major weakness considering that such charge flow governs the operation of batteries.

## Functional characterization using magnetic imaging

Here, we propose to use magnetic microscopy to visualize the buried flow and reactions of electrons and ions during the full battery charge/discharge cycle. Magnetic microscopy encompasses a suite of techniques generally based on measuring stray magnetic fields from a sample or detecting interactions between the magnetization and either electrons or light^41^. We here adopt the perspective of scanning nitrogen vacancy (NV) magnetometry, which combines sensitive detection of magnetic field stray fields at room temperature with nanoscale spatial resolution^57^. A brief discussion on the prospects of other techniques will be provided later.

**Fig. 2:**
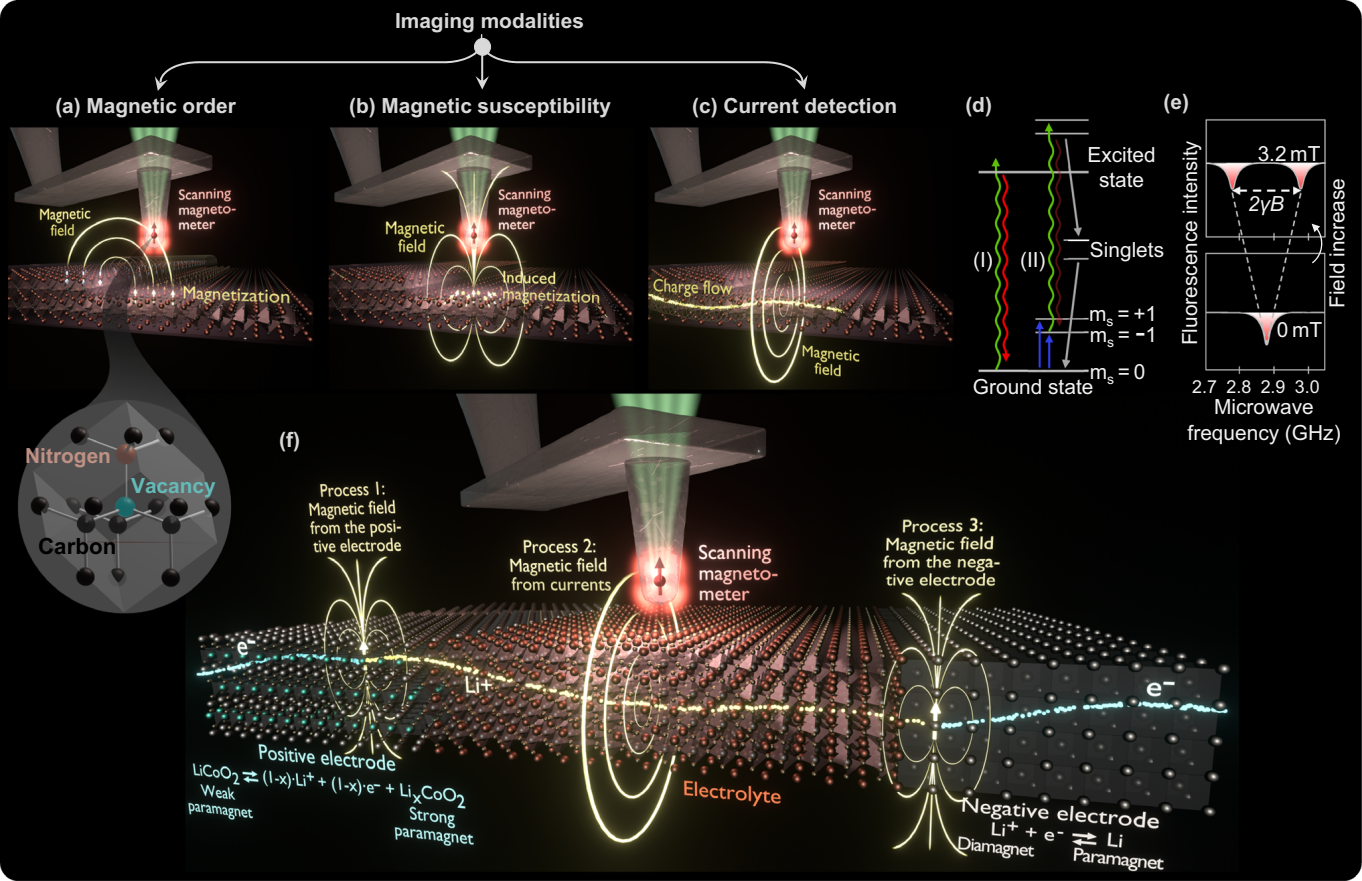
Magnetic microscopy. **a**–**c** Measurement modalities of scanning nitrogen vacancy (NV) magnetometry where the NV sensor is scanned with respect to the sample surface to acquire an image of the magnetic stray field. The stray field is used to infer knowledge about magnetic order, magnetic susceptibility, and current distributions. **d** Simplified electronic structure of the NV where non-radiative processes lead to lower fluorescence intensity of the *m*_*s*_ = ±1 states and to a polarization into the *m*_*s*_ = 0 spin state. **e** Schematics of the fluorescence intensity as a function of microwave frequency. When the frequency is resonant with the *m*_*s*_ = 0 to ±1 transition, two dips with reduced fluorescence split by the Zeeman effect enable the detection of magnetic fields. **f** Schematics of various magnetic field sources in batteries, which can be measured with scanning NV magnetometry, including magnetization at the positive & negative electrodes and ionic & electronic current distributions.

In scanning NV magnetometry, a single magnetically sensitive defect is located $\sim$10 nm from the edge of a diamond scanning probe tip. When the defect is moved relative to the sample, the magnetometric properties of the defects allow for forming a 2D or 3D map of the local magnetic field, which can be used to form quantitative images of the buried current distributions, magnetic order, and magnetic susceptibility of the sample (see Fig. 2a–c)^57,58^. As magnetic fields propagate unperturbed through solid and liquid matter without magnetic order, this magnetic sense allows for seeing buried functional processes in action, including electronic/ionic motion and magnetic changes associated with redox reactions.

The NV defect is formed by replacing two neighboring carbon atoms in the diamond lattice with a nitrogen atom and a vacancy, respectively (see inset in Fig. 2a). This forms the optically and magnetically active electronic structure schematically represented in Fig. 2d. When exposed to green light, the NV defect transitions from the spin ${m}_{s}=0$ triplet ground state to a high-energy excited state, which decays to the ground state by emitting red fluorescent light, as shown in process (I) in Fig. 2d. Microwave radiation with a frequency on the order of a few GHz can be used to excite the ${m}_{s}=0$ spin state to the ${m}_{s}=\pm 1$ spin states. After optical excitation with green light, the resulting excited state has a higher chance of decaying through a metastable singlet state, which does not emit visible light, as depicted by process (II) in Fig. 2d. Hence, a reduction in fluorescence is observed when the microwave frequency is resonant with the energy difference between the ${m}_{s}=0$ and ${m}_{s}=\pm 1$ states. In the absence of a magnetic field, the ${m}_{s}=\pm 1$ states are degenerate, resulting in a single fluorescence dip (Fig. 2e). A magnetic field of magnitude *B* results in the Zeeman effect splitting the ${m}_{s}=\pm 1$ states by $\Delta E=2h\gamma B$ where *γ* ≈ 28 GHz T^-1^ is the gyromagnetic ratio of the NV center and *h* is the Planck constant. This produces two well-defined dips as the microwave frequency is varied. The simplest detection scheme is continuous-wave optically detected magnetic resonance (CW-ODMR) where the field-dependent fluorescence dips are tracked with continuously applied green light and microwaves with varying frequency while scanning the sample underneath the diamond tip. A variety of pulsed measurement schemes based on spin manipulation has also been developed, resulting in greatly improved detection limits^59^.

The atomic size of the NV defect and the favorable defect properties embedded in the diamond host result in an unparalleled combination of high spatial resolution and high sensitivity when employed as a scanning magnetometer^57^. Other NV architectures include sensing the magnetic fields by detecting NVs in a macroscopic slab of diamond in contact with the sample^60^ or dispersing nanodiamonds inside the sample^36^. Employing multiple NV defects in these architectures enables powerful magnetic field imaging over a wide field of view using a camera, rather than generating the image pixel by pixel through raster scanning^61^. However, wide-field imaging typically features a poorer spatial resolution on the order of a micrometer, challenges in ensuring close proximity of the sample to the diamond slab surface, and the invasiveness of interspersing and exciting nanodiamonds within the material. NV magnetometers have already had a transformative impact on understanding processes in electronic and magnetic materials^62–71^, but their use for imaging batteries at the micro- and nanoscale remains elusive. Instead, magnetic characterization of batteries has focused on the macroscale using various magnetic characterization tools^29–35^.

## Magnetic imaging of batteries

In this perspective, we propose a multimodal approach for visualizing the charge and discharge cycle of batteries. In the first imaging mode, the redox reactions at the positive electrode are imaged by the associated magnetic changes when ions and electrons are removed from (added to) the positive electrode during charge (discharge). In mode two, the flow of active ions and electrons is detected by the Ørsted magnetic field formed by the moving charges. In mode three, the redox reactions at the negative electrode are spatially resolved by tracking the transformation of active ions into paramagnetic metals. We provide finite element simulations of a Li|LLZO|Li_x_CoO_2_ solid-state battery, which collectively depict the magnetic stray fields arising from these processes as described in the following sections. Following this, we also discuss the prospects of gaining insight into battery operation and degradation using magnetically ordered phases.

### Positive electrode reactions

**Fig. 3:**
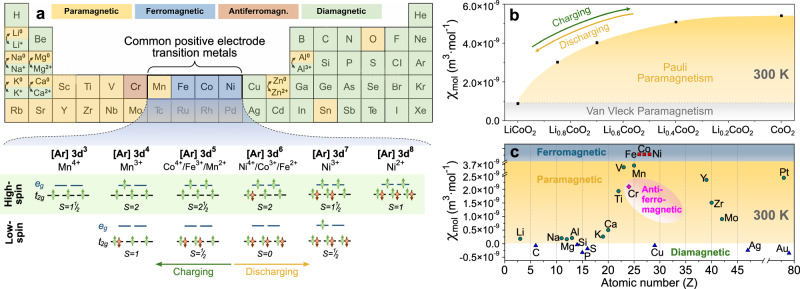
Magnetic periodic table of battery materials. **a** Reduced periodic table depicting the magnetic properties of various elements, including those of selected ions commonly used for batteries. The lower part depicts the low- and high-spin configurations of selected positive electrode transition metals in various oxidation states. The transition metals are assumed to be octahedrally coordinated with the oxygen ions, which is consistent with the case of the layered oxides and LiFePO_4_. **b** Magnetic susceptibility of Li_x_CoO_2_ with different charge states obtained by electrochemically cycling a liquid-state battery with Li_x_CoO_2_ at the positive electrode, as described in Supplementary Section S1. We note that structural and compositional degradation occurs for x <½, which causes additional phases to form beyond Li_x_CoO_2_. **c** Magnetic susceptibility of various elements of relevance to batteries.

Most active materials in the positive electrodes of batteries contain transition metals like Co, Mn, Ni, or Fe, since their multivalent redox states can accommodate the removal/addition of electrons by changing their 3d electron occupancy. In general, one can classify battery materials as 1D, 2D, and 3D ionic conductors with Table 1 outlining common lithium battery materials as well as their structural, electronic, ionic, and magnetic properties at room temperature. Common positive electrode active materials include 1D polyanion oxides such as LiFePO_4_^72^, 2D layered oxides such as LiCoO_2_ and nickel-rich LiNi_x_Mn_y_Co_1-x-y_O_2_, and 3D spinel structures such as LiMn_2_O_4_. As illustrated in Fig. 3a, the transition metals are generally magnetically active owing to the five correlated and energetically accessible 3d states, which promote rich magnetic phase diagrams for these material classes. The magnetic properties are primarily determined by the number of 3d electrons per transition metal atom and the spin arrangement of these electrons. For positive electrodes, the 3d electron occupancy is dictated by the choice of transition metal ion and its redox state. During battery charge and discharge, the redox state of the transition metal ions changes to balance charges after structurally accommodating or releasing lithium. As a consequence, the magnetic properties can be used as a proxy to monitor redox reactions at the positive electrode and state-of-charge through changes in their spin states, see Fig. 3a^73–77^. For the layered oxides and LiFePO_4_, the transition metal ions are octahedrally coordinated with the surrounding ligands, and low- or high-spin configurations form as a balance between the crystal field splitting between *e*_*g*_ and *t*_*2g*_ states and the energy gained by spin alignment (Fig. 3a).

The spin configurations in the positive electrode materials may be affected by a number of additional factors such as polymorphic transitions, distortions in bond angles, and defect formation. This can be leveraged to gain insights into important aspects such as Li-Ni interdiffusion in positive electrodes and lattice distortions^32^, while also complicating interpretation.

For the 2D Li-conducting Li_x_CoO_2_, the lithiation degree is balanced by changing the redox state of the transition metal ion between Co^3+^ and Co^4+^. At room temperature, Li_x_CoO_2_ is paramagnetic at all lithiation stages, but the paramagnetic susceptibility changes significantly with the state-of-charge^73^ as illustrated in Fig. 3b. The experimental procedure, battery cycling curves, and raw magnetometry data behind Fig. 3b are provided in Supplementary Section S1. The lower value of the paramagnetic susceptibility for electronically insulating LiCoO_2_ represents Van Vleck paramagnetism^73^. Upon delithiation, Li_x_CoO_2_ transitions into a Pauli paramagnet at room temperature with a monotonously increasing paramagnetic susceptibility in most of the reversible state-of-charge window (Fig. 3b)^73^. Irrespective of the charge state, the magnetic susceptibility of Li_x_CoO_2_ exceeds that of most elements used in batteries, as outlined in Fig. 3c, which enables the detection and distinction of the Li_x_CoO_2_ charge state in the presence of other elements. Figure 3c also serves as a guide for selecting current collectors and interfacial layers with the lowest magnetic susceptibilities to ensure minimal influence when performing magnetic imaging. We note that Fig. 3c illustrates the magnetic susceptibility of various elements, which is complemented by Table 1 providing the magnetic susceptibilities of battery materials.

**Table 1:** Structural, ionic, electronic, and magnetic properties at 300 K of selected positive electrode, solid electrolyte, and negative electrode materials

	Dimension of Li^+^ conduction [Structure]	Material	Li^+^ diffusivity (cm^2^ s^−1^)	Ionic conductivity (S cm^−1^)	Electronic conductivity (S cm^−1^)	Magnetic state	Magnetic ground state	Molar magnetic susceptibility (m^3^ mol^-1^)	Influence of (de-)lithiation on molar magnetic susceptibility (m^3^ mol^-1^)
**Positive electrode (x = 1)**	1D [Olivine]	Li_x_FePO_4_	10^−14^^129^	10^−4^^130^	10^−9^^130^	Paramagnet^111^	Antiferromagnet^111^	4.5 × 10^−8^^76^	↓ 4.1 × 10^−8^ (x = 0) when fully delithiated^76^
		Li_x_Fe_0.8_Mn_0.2_PO_4_	10^−12^^131^	-	10^−6^^132^	Paramagnet^112^	Antiferromagnet^133^	1.3 × 10^−7^^112^	↓ 8.2 × 10^−8^ (x = 0) when fully delithiated^112^
	2D [Layered]	Li_x_CoO_2_	10^−9^^134^	10^−7^^135^	10^−3^^136,137^	Paramagnet^77^	Paramagnet^33^	1.3 × 10^−9^^77^	↑ 7.2 × 10^−9^ (x = 0.6) when delithiated, plateaus at ∼ 6.0 × 10^−9^ (x < 0.6) on further delithiation^73^
		Li_x_Ni_0.8_Co_0.15_Al_0.05_O_2_	10^−10^^138^	10^−10^^138^	10^−4^^138^	Paramagnet^139^	Paramagnet^139^	3.8 × 10^−8^^139^	↓ 1.5 × 10^−8^ (charged to 4.7 V) when delithiated^139^
		Li_x_MnO_2_	10^−9^^140^	-	10^−8^^140^	Paramagnet^141^	Antiferromagnet^141^	2.7 × 10^−8^^141^	↑ 6.1 × 10^−8^ (x = 0.39) when delithiated^141^
		Li_x_NiO_2_	10^−9^^142^	-	10^0^^143^	Paramagnet^144^	Ferromagnet^144^	2.3 × 10^−8^^144^	↑ 2.8 × 10^−8^ (x = 0.94) when delithiated^144^
		Li_x_Ni_0.33_Mn_0.33_Co_0.33_O_2_	10^−13^^145^ 10^−10^^138^	10^−6^^135^	10^−7^^138^ 10^−6^^135^	Paramagnet^74^	Antiferromagnet^74^	3.1 × 10^−8^^74^	-
		Li_x_Ni_0.5_Mn_0.3_Co_0.2_O_2_	10^−10^–10^−9^^138,146^	10^−3^^135^	10^−3^^135^	Paramagnet^147^	Antiferromagnet^147^	1.6 × 10^−8^^39^	↑ 2.2 × 10^−8^ (x = 0) when fully delithiated^39^
		Li_x_Ni_0.8_Co_0.1_Mn_0.1_O_2_	10^−11^^148^	10^−2^^135^	10^−3^^135^	Paramagnet^149^	Antiferromagnet^149^	2.2 × 10^−8^^149^	-
	3D [Spinel]	Li_x_Mn_2_O_4_	10^−8^^150^	10^−6^–10^−4^^151^	10^−7^-10^−5^^151^	Paramagnet^152^	Antiferromagnet^152^	1.0 × 10^−7^^152^	-
		Li_x_Mn_1.5_Ni_0.5_O_4_	10^−9^^153^	10^−9^^153^	10^−6^^153^	Paramagnet^154^	Ferromagnet^154^	1.4 × 10^−7^^154^	-
**Negative electrode (x = 0)**	3D [Spinel]	Li_4+3x_Ti_5_O_12_	10^−11^^155^	10^−5^^156,157^	10^−6^^158^	Paramagnet^155,159^	Paramagnet^155,159^	4.1 × 10^−10^^159^	↑ 1.3 × 10^−8^ (x = 0.95) when fully lithiated^159^
	3D [Hexagonal]	Li_x_C_6_ (Graphite)	10^-5^^160^	-	10^4^^161^ (Single crystal)	Diamagnet^162^	Diamagnet^162^	-5.0 × 10^−9^^162^	↑ 1.2 × 10^−9^ (x > 0.5) when lithiated^162^ 0.34 <x < 0.5: Transition to Paramagnet^162^
	3D [Diamond]	Si	10^−9^^163^	10^−4^^163^	10^−4^^163^	Diamagnet^164^	Diamagnet^164^	-4.2 × 10^−11^^164^	-
	3D [Tetragonal]	SnO_2_	10^−14^^165^	-	10^0^^166^ (Single crystal)	Diamagnet^167^	Diamagnet^167^	-7.3 × 10^−10^^167^	-
	3D [Cubic]	Co_3_O_4_	10^−9^^168^ (Microsphere)	-	10^−3^^169^ (Thin film)	Paramagnet^170^	Antiferromagnet^170^	7.8 × 10^−8^^171^	-
**Electrolyte**	2D [Layered] (Perovskite)	Li_0.3_La_0.56_TiO_3_ (LLTO)	10^−8^^172^	10^−3^^172^	10^−9^^173^ 10^−7^^174^	Paramagnet^175^	-	-	-
	3D [NASICON]	Li_1.3_Al_0.3_Ti_1.7_(PO_4_)_3_ (LATP)	10^−10^^176^	10^−3^^176,177^	10^−8^^178,179^	-	-	-	-
		Li_1.5_Al_0.5_Ge_1.5_(PO_4_)_3_ (LAGP)	10^−9^^180^	10^−4^^181,182^	10^−8^^183^	-	-	-	-
	3D [Tetragonal] (Garnet)	Li_7_La_3_Zr_2_O_12_ (LLZO)	10^−14^^184^	10^−6^^185^	10^−7^^186^	Diamagnet	-	-1.2 × 10^-11^ (Measured here)	-
	3D [Cubic] (Garnet)	Li_6.25_(Ga,Al)_0.25_La_3_Zr_2_O_12_	10^−9^-10^−8^^187,188^	10^−4^^189–192^	10^−8^ (Al)^189^ 10^−7^ (Ga)^190^	Diamagnet^193,194^	-	-	-
		Li_6.25_Fe_0.25_La_3_Zr_2_O_12_	-	10^−5^^195^ 10^−3^^196^	10^−6^^195^	Paramagnet^109^	-	-	-
	Nonlinear [Oxynitride glass]	Li_3_PO_4_ (Thin film)	10^−12^^197^	10^−8^^198^ 10^−7^^199^	10^−10^^199^	-	-	-	-
		Li_2.4_PO_2.2_N_0.6_ (LiPON) (Thin film)	10^−9^^200^	10^−6^^200^	10^−13^^200^	-	-	-	-

Unless specified differently, bulk values are reported.

**Fig. 4:**
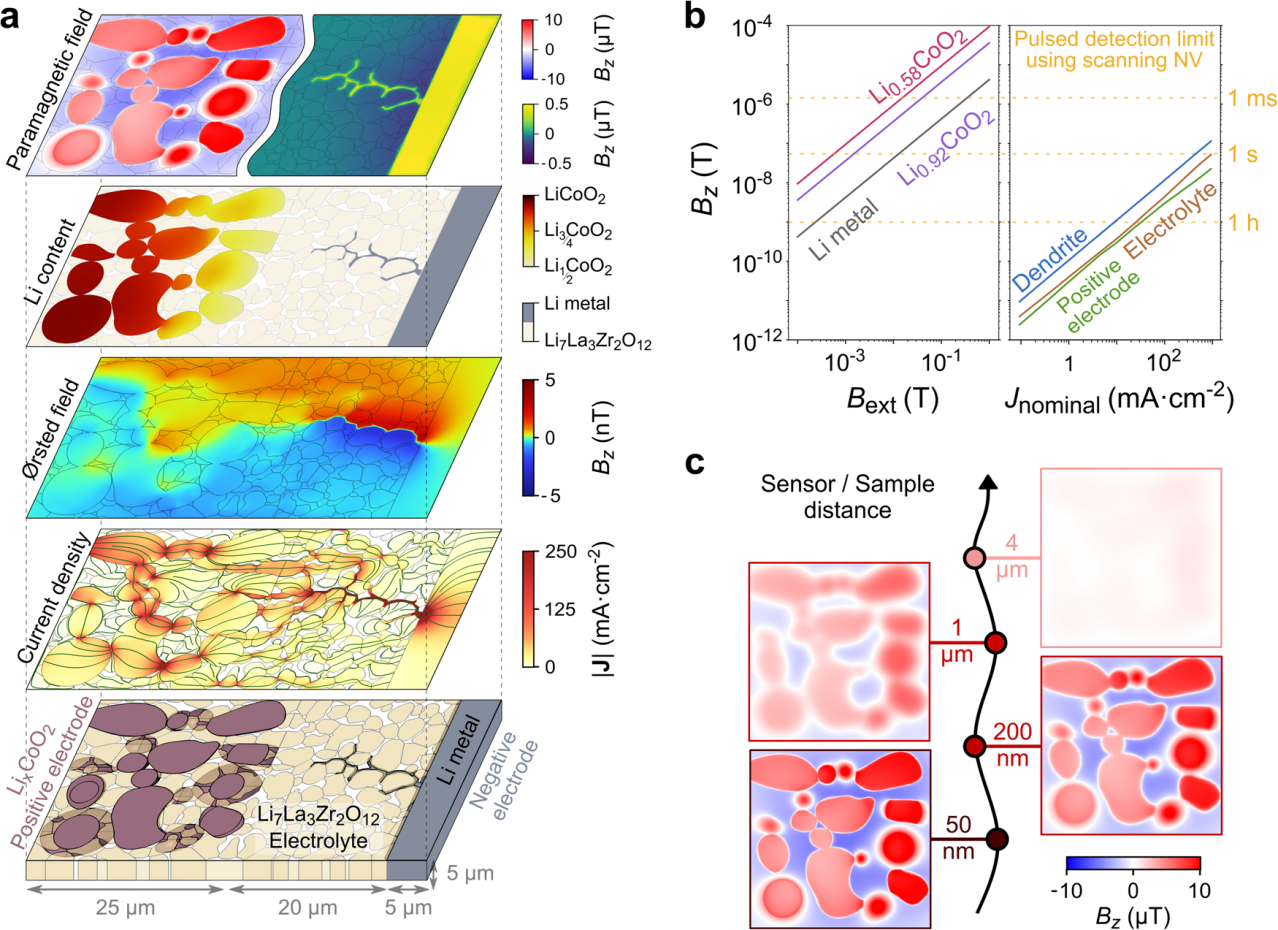
Multimodal magnetic microscopy on batteries. **a** Finite element simulations of a Li|Li_7_La_3_Zr_2_O_12_|Li_x_CoO_2_ solid-state battery with the Li_x_CoO_2_ positive electrode particles containing various lithiation states and a lithium metal dendrite forming in the Li_7_La_3_Zr_2_O_12_ electrolyte grain boundaries. The geometry is inspired by experimental studies^81,82^ as described in Supplementary Section S3. Here, both the dendrite and part of the positive electrode particles are buried below the surface, as shown in the lower schematics. In the middle panels, we show the magnitude of the local current density ( | J | ) with 23.5 mA·cm^−2^ nominal current applied as well as the out-of-plane component of the Ørsted magnetic field (*B*_*z*_) the current produces 50 nm above the surface. In the upper two panels, we show the lithium metal locations and the lithium content of the Li_x_CoO_2_ particles in addition to the magnetic field produced by the paramagnetic components in an external field of 100 mT. **b** The simulated signal strengths and magnetometry detection limits as a function of the external magnetic field (*B*_ext_) for the paramagnetic lithium metal and Li_x_CoO_2_ and the nominal current density (*J*_nominal_) for the Ørsted magnetic field. **c** Magnetic stray fields from the Li_x_CoO_2_ particles at different distances between the nitrogen vacancy (NV) sensor and the surface of the positive electrode.

NV magnetometry allows for imaging the local magnetic susceptibility in the presence of an external magnetic field using stray field imaging (process 1 in Fig. 2f), thus spatially resolving the redox process at the positive electrode. In Fig. 4a, we provide finite element calculations^78^ of the electrochemical processes and their associated magnetic stray field from a Li|LLZO|Li_x_CoO_2_ solid-state battery. Here, LLZO fills the gaps between the Li_x_CoO_2_ particles to promote redox homogeneity, but conductive carbon additives are not included in the simulations owing to the high electronic conductivity of Li_x_CoO_2_^79,80^. The configuration modeled corresponds to cross-sections of conventional solid-state batteries with electrodes on the top/bottom of an electrolyte or in-plane solid-state batteries with ionic transport taking place in the plane of an electrolyte thin film. Supplementary Section S2 describes the model in detail, including its experimental input parameters and geometrical inspiration from X-ray tomography and scanning electron microscopy studies^81,82^. We find the redox reactivity to be homogeneous when electrochemically charging the battery at low and moderate charge rates of C/10 and 1 C, which corresponds to nominal current densities of 0.09 and 0.94 mA·cm^-2^, respectively (Supplementary Section S2.5). However, at fast charge rates of 5 C (4.70 mA·cm^-2^), we observe a large gradient in the lithium concentration where the positive electrode particles closest to the negative electrode are delithiated faster, as displayed after 4 minutes of charging in the second panel of Fig. 4a where Li_x_CoO_2_ remains in the reversible operating range (x > ½). The ionic conductivity of Li_x_CoO_2_ varies non-monotonically by more than an order of magnitude depending on the lithium content, but the lithium-ion diffusion remains sufficiently high to promote a relatively homogeneous lithium composition within each Li_x_CoO_2_ particle. Such redox heterogeneity is commonly observed for various positive electrode active materials^6,7,24,48^ and is consistent with scanning transmission X-ray microscopy revealing interparticle variations in the lithium content upon a fast 4 C delithiation in a liquid-state battery with Li_x_(Ni_1/3_Mn_1/3_Co_1/3_)O_2_ at the positive electrode^7^.

Applying a sample-wide magnetic field of 100 mT perpendicularly to the Li_x_CoO_2_ surface induces a stronger magnetization in delithiated regions compared to lithium-rich regions, effectively resulting in a magnetic stray image revealing the underlying state-of-charge (top panel of Fig. 4a). Although this is superimposed with shape effects, the trend is even observed in positive electrode particles buried several hundreds of nanometers below the surface layer such as the lower, right particle in Fig. 4a. The stray field is calculated at a plane located 50 nm above the battery surface, which is consistent with typical NV stand-off distances. Supplementary Fig. S10 displays the results for the external magnetic field and the NV quantization axis both being perpendicular, parallel, or tilted 54.7° with respect to the sample surface, corresponding to diamond tips with common crystallographic terminations. Although the simulated magnetic stray field differs significantly depending on the external field and NV orientation, the stray field is on the order of 10 µT in all cases, which is detectable with CW-ODMR sensitivities of $\sim$1 µT·Hz^−0.5^ and the pulsed detection limits of $\sim$50 nT using scanning NV magnetometry with 1 second integration time^58,63,83,84^. The paramagnetic moments are unidirectional and aligned along the external magnetic field axis, which simplifies the reconstruction of the underlying magnetization from the measured magnetic field images. If the magnetization can be assumed to be 2-dimensional, it can be constructed uniquely from the measured stray magnetic field^85^, otherwise, the reconstruction becomes more involved^41,86,87^ and structural knowledge, such as particle sizes and shapes, can be an advantage. Owing to the paramagnetic properties of Li_x_CoO_2_ in the positive electrode, the magnetic signal strength scales linearly with the applied magnetic field (Fig. 4b), which provides both the possibility of enhancing the signal strengths with larger fields applied using superconducting or permanent magnets as well as performing local ac susceptometry by delivering smaller alternating magnetic fields using the microwave antenna^58^. The linearity can further serve to establish the paramagnetic origin of the measured magnetic stray fields. We note that, as overviewed in Table 1 and Supplementary Section S1, many positive electrode materials change their magnetic susceptibility as a function of lithium content, which adds to the prospects of visualizing the local state-of-charge in positive electrode materials beyond Li_x_CoO_2_.

### Charge flow

Following the redox reaction at the positive electrode, the electrons flow to the negative electrode through the electrodes and external circuit, whereas ions flow to the negative electrode through the electrolyte. The electronic and ionic currents produce an Ørsted magnetic field (process 2 in Fig. 2f), which is simulated in Fig. 4a. The simulations are performed with a high input current density of 23.50 mA·cm^−2^ (charge rate of 25 C) as discussed later. The current redistributes near key defects such as lithium dendrites, void, grain boundaries, and chemical inhomogeneities^88^, which leads to distinct imprints in the stray field. The high electronic conductivity of the lithium metal dendrite focuses most current within the electrolyte in a narrow region, which eventually leads to battery short-circuiting if the dendrite extends to the electronically conducting positive electrode. At the positive electrode, the current distributes in response to the higher ionic conductivity in LLZO compared to Li_x_CoO_2_, the distribution of grains and grain boundaries, and how the heterogeneous lithium content impacts the highly lithium-dependent ionic conductivities in Li_x_CoO_2_. The current gradually becomes more focused in the positive electrode and electrolyte as it approaches the dendrite. In the absence of a conductive carbon matrix, electronic current hot spots are formed at the finite-sized contact points between Li_x_CoO_2_ particles, while ionic current hot spots form in interconnected LLZO particle chains connecting the extended tips of the dendrite. With a nominal current density of 23.50 mA·cm^−2^, the magnetic signal strengths associated with this battery geometry are on the order of 1–5 nT, with larger signals around the dendrite and smaller signals in inactive battery regions.

Two key advantages of imaging this Ørsted magnetic field are that it directly reveals which defects and local features are important for the battery operation and that the buried current density can be quantitatively reconstructed from the measured stray field during operation^85,89^. If the current can be approximated to flow in 2D, it can be uniquely reconstructed using an inverse filter method^85,89^, whereas more complex 3D current distributions require inverse modeling^41,90,91^. The magnetic signal strength from the inhomogeneous currents at the dendrite, electrolyte, and positive electrode is presented in Fig. 4b as a function of the nominal current density and compared with NV magnetometry sensitivities^83,92^. The magnetic signals can be strengthened by increasing the current, but the application of high currents is generally limited by the growth of lithium dendrites above a critical current density^93^. Typical critical current densities in solid-state batteries range from 0.1 to a few mA·cm^−2^ with a 10-fold increase after negative electrode|electrolyte interface optimization^5,94–96^. In a recent report, a new high-throughput method for characterizing the critical current density has been proposed and tested on ceramic electrolytes withstanding current densities exceeding 300 mA·cm^−2^ ^97^. At the local scale, extremely large current densities exceeding 10,000 mA·cm^−2^ have been reported near lithium dendrites^5^. Longer sample acquisition can be used to resolve the weak Ørsted magnetic fields by preparing the battery in a desired state using direct currents while probing the current distribution using alternating currents. The critical alternating current density is generally inadequately investigated in solid-state batteries, but studies have revealed that alternating currents can improve the performance by mitigating dendrite formation or preheating the battery^98–102^. Here, the use of alternating currents greatly increased the critical current density compared to direct currents, resulting, for instance, in a more than ten-fold increase when increasing the frequency from 0.1 to 100 Hz^101,102^. Although more work is needed to elucidate the alternating critical current densities, nominal alternating currents on the order of up to 10-100 mA·cm^−2^ may be expected in solid-state batteries, with higher values obtainable after employing strategies for enhancing the critical current density^93,103^. The local current densities near dendrites are expected to be 100–10,000 mA·cm^−2^. This results in measurement integration times ranging from sub-seconds to tens of minutes per pixel to resolve these currents with scanning NV magnetometry (Fig. 4b). The measurements can be further accelerated using more advanced protocols such as spin memory-based readout sequences, which have been demonstrated to yield a 10-fold improvement in the sensitivity of single and ensemble NVs^104,105^.

As illustrated in Supplementary Section S2.5, the magnetic signals associated with sourcing currents through only the electronically conducting electrode are 4 orders of magnitude larger than the ionically limited currents simulated in Fig. 4a. The electronic and ionic contributions to the magnetic field may be discriminated by performing temperature-dependent measurements to thermally activate or reduce ionic transport, probe the transport in various frequency regimes, or employing ion- or electron-blocking electrodes^106^. Further exploration of these possibilities could uncover the individual contributions of buried electronic and ionic currents at the nanoscale, which is generally not possible with conventional analytic tools. This holds great importance not only for understanding the role of electronic leakage in electrolyte grain boundaries for forming dendrites^21,107^, but also for probing the nanoscale impact of solid-electrolyte interphases and inter-/intraparticle electronic conduction in positive electrodes.

### Negative electrode reactions

The use of pure metals as negative electrodes greatly increases the energy density, particularly in anode-free architectures where the negative electrodes are formed by battery cycling rather than during fabrication. When employing metals at the negative electrodes, the reduction of most active ions used for charge transport in lithium and post-lithium batteries concurs with a transition to paramagnetic pure metals. As illustrated in Fig. 3, this includes the majority of monovalent (Li^+^, Na^+^, K^+^), divalent (Mg^2+^, Ca^2+^), and trivalent (Al^3+^) ions.

Applying an external magnetic field of 100 mT produces a magnetic stray field from the paramagnetic lithium metal, which can be used to directly image the local state-of-charge, dendrites, and lithium metal inclusions in the diamagnetic LLZO electrolyte, as shown in Fig. 4a. Due to the stronger magnetic stray field from Li_x_CoO_2_ compared to the Li metal, the paramagnetic fields from the lithium metal and Li_x_CoO_2_ are simulated separately and plotted with two color bars. Similar visualization of dendrites and inactive metal inclusions is valid for the paramagnetic metals Li, Na, K, Mg, Ca, and Al present in diamagnetic electrolytes. To date, the magnetic properties of only a few solid electrolytes have been reported (see Table 1). In general, most electrolytes lack 3d transition metal ions, whereby it is reasonable to hypothesize that the majority exhibits diamagnetic characteristics based on their expected spin states^108^. However, Fe dopants and oxygen vacancies have been observed to induce paramagnetism in LLZO^109,110^, and hence, we encourage the field to further characterize the magnetic properties of electrolytes to form a solid future basis for visualizing metallic inclusions.

The signal strength as a function of the applied magnetic field is shown in Fig. 4b and compared with the detection limit of NV magnetometers. Although the predicted signals are one order of magnitude weaker for lithium metal compared to Li_x_CoO_2_, their detection remains feasible in high external magnetic fields. Similar to the induced magnetization of the positive electrode, quantitative reconstruction of the underlying magnetization from the measured magnetic field images is also simplified by the unidirectional magnetization. Furthermore, the magnetic field from the magnetization non-perturbatively penetrates nonmagnetic protective layers and thin current collectors, which makes it possible to visualize redox processes at the negative electrode even when the air-sensitive pure metal negative electrodes are covered by protective layers.

### Magnetically ordered phases

Most battery materials are considered either paramagnetic or diamagnetic at room temperature, but magnetically ordered phases may emerge particularly in two cases: First, as outlined in Table 1 (magnetic ground state), many positive electrode active materials order magnetically below transition temperatures on the order of tens of Kelvin. The characteristics of the magnetic order, including the transition temperature^111,112^, are sensitive to the lithium content and crystalline phase. Scanning magnetometry measurements performed below or in the vicinity of the transition temperature can introduce enhanced signal strength and spatial contrast in the magnetic stray field arising from spatial inhomogeneities affecting the ferromagnetic order or magnetic susceptibility. Cooling further freezes the battery in its present state, allowing for long integration times.

Second, imperfections, defects, and degradation products arise during battery synthesis or cycling, which can produce magnetically ordered states. Key examples include (1) the recent operando detection of Fe_3_O_4_ and Fe, which represents an impurity in LiFePO_4_^36^, (2) Li-Ni interdiffusion in LiNi_x_Mn_y_Co_1-x-y_O_2_, whereby Ni located in the lithium layers can ferromagnetically couple two adjacent transition metal layers^32,74^, and (3) degradation products such as the degradation of Li_x_CoO_2_ into magnetically ordered Co_3_O_4_ and CoO at high voltages and delithiation^15,113,114^.

## Prospects and limitations of NV magnetometry for imaging batteries

**Fig. 5:**
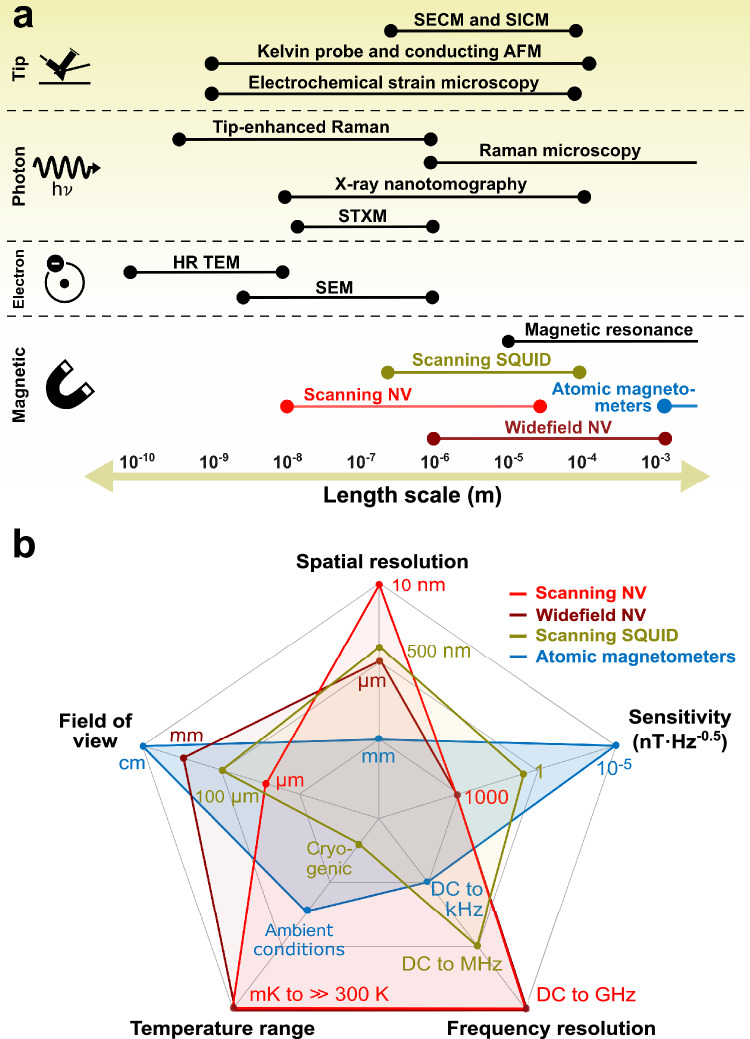
Characteristics of magnetic microscopy techniques. **a** Characteristic spatial resolution and typical field-of-view in high-resolution mode for selected imaging techniques used to characterize batteries. **b** Comparison between commercially available magnetic imaging techniques, including scanning nitrogen vacancy (NV) magnetometers, wide-field NV magnetometers, atomic magnetometers, and scanning superconducting quantum interference devices (SQUIDs). The data forming the basis for this figure is provided in Supplementary Section S3 along with the relevant references.

NV magnetometry may complement established nano- and microscale imaging techniques for battery characterization, as outlined in Fig. 5a, with more information provided in Supplementary Section S3. In contrast to other imaging techniques, NV magnetometry provides a non-destructive view into the functional properties such as current imaging with sub-surface sensitivity. Scanning NV magnetometry is typically done with a fixed separation of several tens of nanometers between the sample surface and the sensor. However, larger distances to the NV sensor occur if the magnetic field sources are buried underneath current collectors, electrodes, or protective layers. In contrast to, e.g., transmission microscopy and most other scanning probe techniques, the magnetometer can detect features buried several micrometers into the battery, although the signals are weaker and resolved with a poorer spatial resolution on the order of the sensor/source distance (Fig. 4c). However, lacking the extended depth resolution of X-ray techniques, NV magnetometry is expected to support primarily the ex-situ characterization of the ionic, electronic, and magnetic properties of individual battery components as well as in-situ measurements on battery cross-sections, thin-film batteries, or exposed surfaces such as electrode surfaces. Interspersing nanodiamonds in the battery matrix may, however, provide an exception to this as recently demonstrated^36^, although the transmission of optical light and microwave to the battery interior, the dedicated sample synthesis, and the invasiveness may provide limitations hereof.

The practical realization of NV magnetometry depends on the NV architecture employed, but in all cases, the invasiveness should be investigated and potentially eliminated by optimizing the measurement protocol, particularly when performing operando imaging. For scanning NV magnetometry, an exposed surface with an acceptable roughness on the order of a micrometer and optional electrical connections are needed. Determining only the magnetic state of the individual battery materials is, hence, readily accessible without dedicated sample preparation beyond surface polishing. Advancing to current imaging, an important limitation is that scanning magnetometers are only sensitive to currents parallel to the surface, thus emphasizing the importance of cross-sectional or in-plane geometries as in Fig. 4a. When interspersing nanodiamonds into the battery matrix, care must be taken to minimize the invasiveness of the foreign material and avoid high temperatures in the sample synthesis. Lastly, when employing wide-field imaging, the rigid diamond surface and the sample need to be in close proximity, which can be realized by preparing the battery materials directly on diamond substrates, transferring the battery materials to the diamond, or placing small diamond pieces on the battery surface. Mature transfer techniques of van der Waals materials can further be employed when using spin defects such as boron nitride vacancy centers in few-layer hexagonal boron nitride for magnetometry, which can also benefit from reduced sensor-to-source distances^115–117^.

The subsurface sensitivity to the local distributions of currents and magnetization is inherited from probing the stray magnetic fields, which can be achieved using a range of techniques^41^. Figure 5b provides a comparison of selected, commercially available techniques, including scanning superconducting quantum interference devices (SQUIDs), wide-field and scanning NV magnetometers, and atomic magnetometers (see Supplementary Section S3 for further information). The improved detection limits of SQUIDs and atomic magnetometers are particularly advantageous when resolving the weak magnetic fields from batteries, but the low operating temperature of SQUIDs and the poor spatial resolution of atomic magnetometers constrain their application. NV magnetometers advantageously combine room temperature operation with nano- or microscale imaging resolution. The lower sensitivity of CW-ODMR provided in Fig. 5b can be improved using pulsed protocols and long integration times, particularly when employing wide-field NV magnetometry where the entire image is acquired simultaneously.

An additional prospect of using NV magnetometry is that it is a multifunctional sensing platform with a plethora of detection modes, which enable powerful correlated imaging. Beyond the present focus on visualizing the current flow and magnetic states associated with redox reactivity and defects, other imaging modalities can provide complementary information, including:

1. The simultaneous acquisition of surface topology changes detected with scanning NV magnetometry, which can provide information about crack formation, particle sizes, and strain evolutions.
2. Optical operando microscopy acquired by periodically focusing on the sample rather than the diamond, which can detect redox progression in graphite negative electrodes, lithium plating, and cracks^118^.
3. Local temperature imaging sensed by measuring the temperature-dependent energy difference between the *m*_*s*_ = 0 and *m*_*s*_ = ±1 states^119,120^, which can give insights into the formation of thermal hot spots and dendrite formation that are essential for mitigating thermal runaway.
4. Electric field imaging detected by either sensing the electric field-dependent energy difference between the *m*_*s*_ = 0 and *m*_*s*_ = ±1 states or leveraging the electric field-dependent transitions away from the bright NV^−^ state^119,121^, which may complement current imaging with associated insights into the electric field distribution. The effect of the temperature and electric field variations can be distinguished from magnetic field variations as the former shifts both *m*_*s*_ = ±1 states with respect to the *m*_*s*_ = 0 state whereas the magnetic fields split the *m*_*s*_ = ±1 states.
5. Magnetic resonance measurements where the NV sensor is used to magnetically detect electron or nuclear spins^42,122,123^. This can provide a wealth of information, including chemical identification of the magnetic field sources, local measurements of the lithium diffusion, and localization of lithium metal, as established with conventional electron paramagnetic and nuclear paramagnetic resonance^37,38,124,125^.

The possibility of correlating local imaging of charge dynamics with the chemical and mechanical state of the battery may help determine how heterogeneous redox states, crack propagations, and undesired lithium inclusions locally affect the battery performance. Here, the susceptometry mode enables locating lithium metal and discriminating between active and inactive lithium, which can be correlated with the current imaging mode to reveal the current-active parts of the battery and dendrite-induced current focusing as depicted in Fig. 4a. Combining these modalities with simultaneously acquired changes in the topography and optical appearance induced by cracks may unambiguously resolve whether lithium dendrites initiate and grow in fracture points starting from the electrode|electrolyte interface^5,126,127^ or grow by connecting inactive lithium metal inclusions in the electrolyte^21,128^.

In conclusion, magnetic imaging and particularly NV magnetometry may find diverse uses as a new probe for correlated and non-correlated visualization of functional processes in lithium and post-lithium batteries. The methods can bring new insights into redox reactions at the positive and negative electrode and degradation pathways while probing the effect on the battery charge dynamics and current distributions.

## Supplementary information

Supplementary Information

## Data Availability

All data needed to evaluate the conclusions in the paper is present in the paper and/or the Supplementary Information. The source data for Figs. 3b, 3c, 4b, and S1–S4 is available at 10.11583/DTU.28152410. Additional data is available on request.
