# Supplementary material for: Magnetic microscopy for operando imaging of battery dynamics
Source: Nat Commun. 2025 Sep 17;16:8303. doi: 10.1038/s41467-025-63409-y (PMC12443997; doi:10.1038/s41467-025-63409-y)
Supplement: Supplementary file 1 — Supplementary Information [file 41467_2025_63409_MOESM1_ESM.pdf]

Magnetic microscopy for operando imaging of battery dynamics

Supplementary Information

Stefan Pollok[1], Mohamad Khoshkalam[1], Fardin Ghaffari-Tabrizi[1], Fran Kurnia[2], Danni Wang[2], Siqi Li[2,3], Dominik B. Bucher[2], Jennifer L. M. Rupp[2,3], Dennis V. Christensen[1,4]

[1] *Department of Energy Conversion and Storage, Technical University of Denmark, Kongens Lyngby, Denmark*
[2] *Department of Chemistry, School of Natural Sciences, Technical University of Munich, Garching, Germany*
[3] *TUMint.Energy Research GmbH, Garching, Germany*
[4] *Institute for Advanced Study, Technical University of Munich, Garching, Germany*

Contents

S1 Magnetic susceptibility of Li_xCoO_2 2
S1.1 Positive electrode sample preparation 2
S1.2 Li-ion battery assembly and testing 2
S1.3 Galvanostatic charging curve of Li-ion battery cell 3
S1.4 Magnetic susceptibility measurement at various lithiation states . 4

S2 Simulations - Solid-state Li-ion battery model 6
S2.1 Geometrical setup 6
S2.2 Input parameters and references 7
S2.3 Modeling approach 8
S2.4 Procedure to obtain state-of-charge of Fig. 4 in the main text . . 10
S2.5 Lithium concentration after charging with C/10, 1C, and 5C rate 11
S2.6 Paramagnetic field from different NV axis orientations 15

S3 Characteristics of magnetic microscopy techniques 15
S3.1 Overview characterization methods 16
S3.2 Benchmark of magnetic imaging techniques 17

Email address: `dechr@dtu.dk` (Dennis V. Christensen)

S1. Magnetic susceptibility of Li_xCoO_2

To determine the molar magnetic susceptibility χ_{mol} of lithium cobalt oxide (Li_xCoO_2) at various lithiation states x, the following experimental procedures were performed as described in this section.

S1.1. Positive electrode sample preparation

To prepare the positive electrode samples, commercially available lithium cobalt oxide powder (MSE PRO Lithium Cobalt Oxide $LiCoO_2$) was mixed with polytetrafluoroethylene powder (PTFE - Sigma-Aldrich) in a 9:2 or 1:1 weight ratio. This ratio was chosen to achieve a balance between high mechanical strength and electrochemical performance. All powders were initially stored in a glovebox (<0.1 ppm O_2, <0.1 ppm H_2O), and were later removed for sample preparation. The mixed powder was milled in air without any solvent or dispersant for 24 hours at 200 rpm in a polyethylene bottle using zirconia beads as the milling medium. The resulting powders were uniaxially pressed into disks (10 mm in diameter and approximately 0.5 mm in thickness) under a load of 40 kN. The samples were then returned to the glovebox to dry for 48 hours before being assembled into a Swagelok cell.

S1.2. Li-ion battery assembly and testing

The prepared positive electrode disks were assembled into a Swagelok cell as the working electrode, using a PFA sleeve and stainless steel pistons as current collectors. The $LiCoO_2$ disk working electrodes were separated from the Li-foil counter/reference electrode by a glass fiber separator. A liquid electrolyte, consisting of a 1M solution of $LiPF_6$ in a 1:1 volume ratio of ethylene carbonate (EC) and dimethyl carbonate (DMC), was added to complete the cell assembly. All assembly procedures were conducted inside a glovebox (<0.1 ppm O_2, <0.1 ppm H_2O). The assembled cells were then charged under galvanostatic conditions at charge rates of C/10 (1:1 ratio), C/200 (9:2 ratio), and C/400 (9:2 ratio), calculated based on the theoretical specific charge capacity of 274 mAh/g, with a cut-off voltage of 4.7 V at 40° C, using a Biologic VMP-300 potentiostat. The low C-rates, such as C/200 and C/400, were selected to ensure more homogeneous delithiation of the Li_xCoO_2 structure during the charging process. To obtain Li_xCoO_2 samples with varying lithium contents, the cells were charged at C/200 rate and C/400 rate to different states of charge, each corresponding to a specific lithium content x in Li_xCoO_2 composition, within the range of $0 \leq x \leq 1$.

It was assumed that the theoretical specific charge capacity of 274 mAh/g corresponds to a fully delithiated state ($x = 0$). The x values for other samples were estimated based on this assumption, and the specific charge capacity to which the cells were charged. After reaching the predefined state-of-charge, the Swagelok cell was disassembled inside the glovebox. The delithiated positive electrode disk was removed, rinsed with the same liquid electrolyte, and then dried inside the glovebox for 48 hours at room temperature.

S1.3. Galvanostatic charging curve of Li-ion battery cell

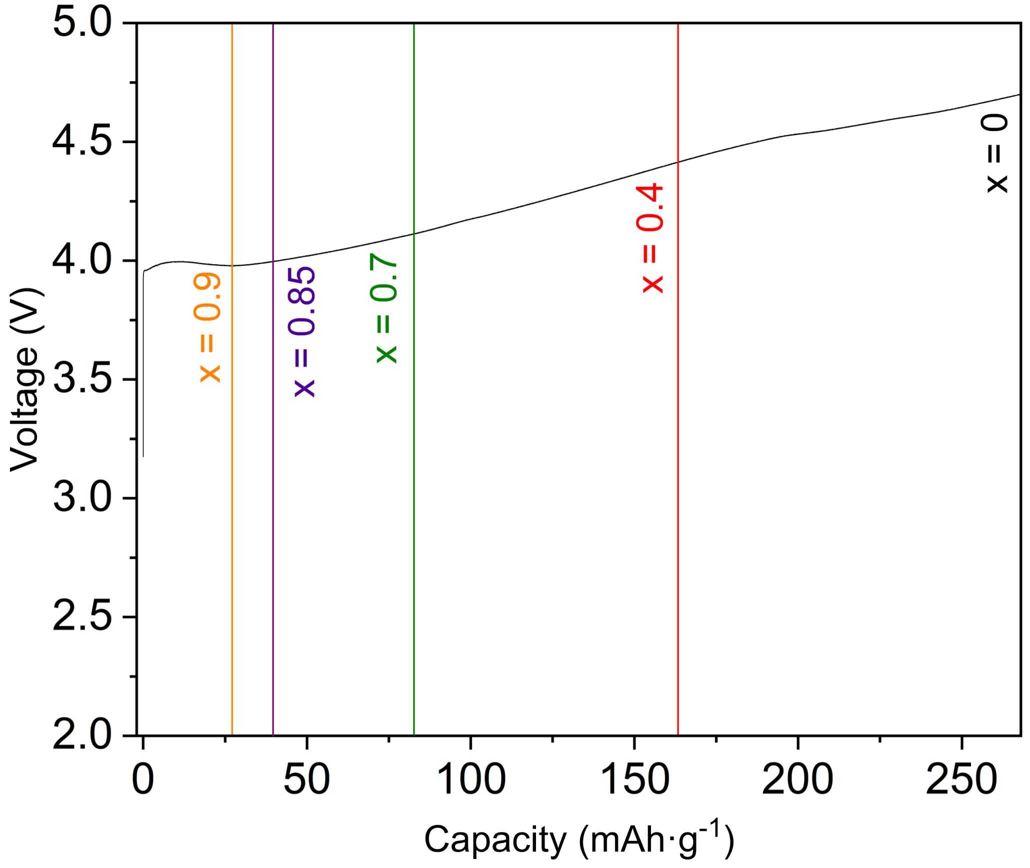

Figure S1: **Galvanostatic charging curve of a Li-ion battery cell, at C/10 rate for complete delithiation after 10 hours.** The specific charge capacities, in which the Li_xCoO_2 samples were selected (at charge rates of C/200 and C/400) for vibrating sample magnetometer measurements, are demonstrated using vertical lines.

S1.4. Magnetic susceptibility measurement at various lithiation states

The magnetic moment of Li_xCoO_2 at various delithiated states was measured under various fields from -2 to 2 T at room temperature using a vibrating sample magnetometer system (Lake Shore Cryotronics VSM 7407). The resulting curves are presented in Fig. S2. The molar magnetic susceptibility of the samples was calculated in SI units from the slope of the linear fit to the M-H curve: χ_{mol} = 0.9 (x = 1), 3.0 (x = 0.85), 4.0 (x = 0.7), 5.1 (x = 0.4), 5.4 (x = 0) $[\times \mathbf{10^{-9}\ m^3 \cdot mol^{-1}}]$. These magnetic susceptibility values follow the same trend and are in good agreement with data known from literature [1, 2].

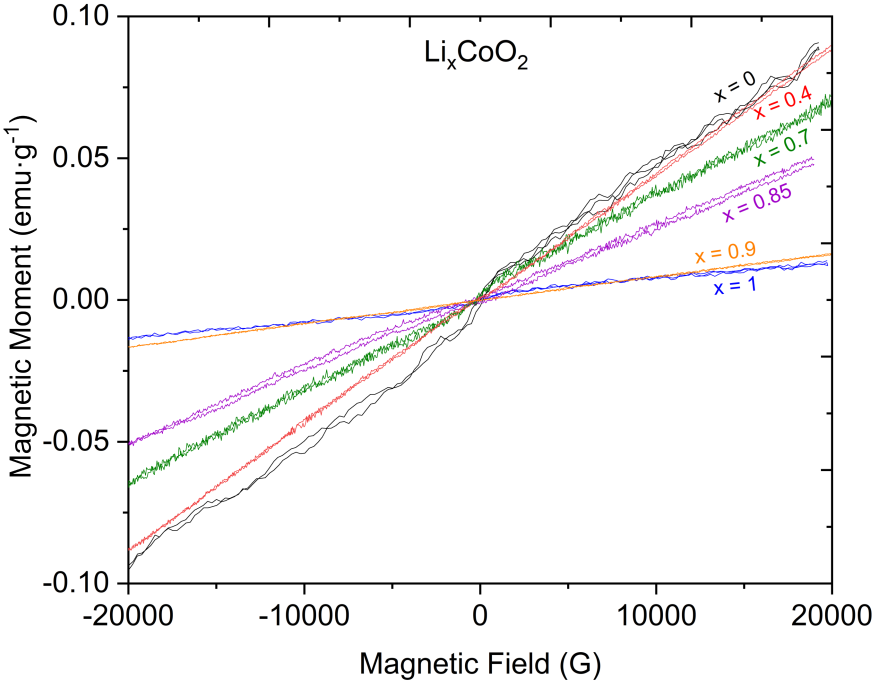

Figure S2: **Magnetization curves of Li_xCoO_2 samples with varying lithium content x at room temperature.** The magnetic susceptibility is found from the slope of the linear fit to these curves. It can be seen that the magnetic susceptibility increases with decreasing lithium content.

The variation of magnetic susceptibility of other popular positive electrode materials, such as $Li_{1.02}Ni_{0.50}Mn_{0.29}Co_{0.19}O_2$ (NMC532) and $LiFePO_4$ (LFP), at different states of charge is demonstrated in Fig. S3 and Fig. S4, respectively [3, 4]. It should be noted that in NMC532, the magnetic susceptibility varies at different states of charge by the change in lithium content within the crystal structure, and higher lithium content is associated with higher susceptibility [3]. In LFP, on the other hand, by increasing the state-of-charge, $LiFePO_4$ with high magnetic susceptibility transforms into a less magnetic $FePO_4$ phase [4]. Therefore, high states of charge are associated with two-phase mixtures of lower magnetic susceptibility.

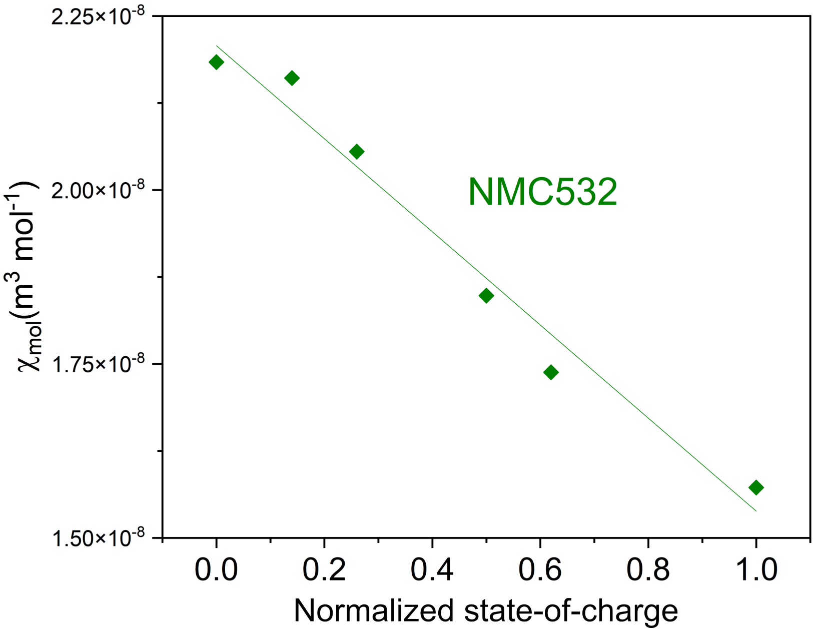

Figure S3: **Molar magnetic susceptibility of NMC532 measured at different states of charge.** The apparent variation in χ_{mol} is associated with different lithium content within the crystal structure of NMC532. Data from Ref. [3].

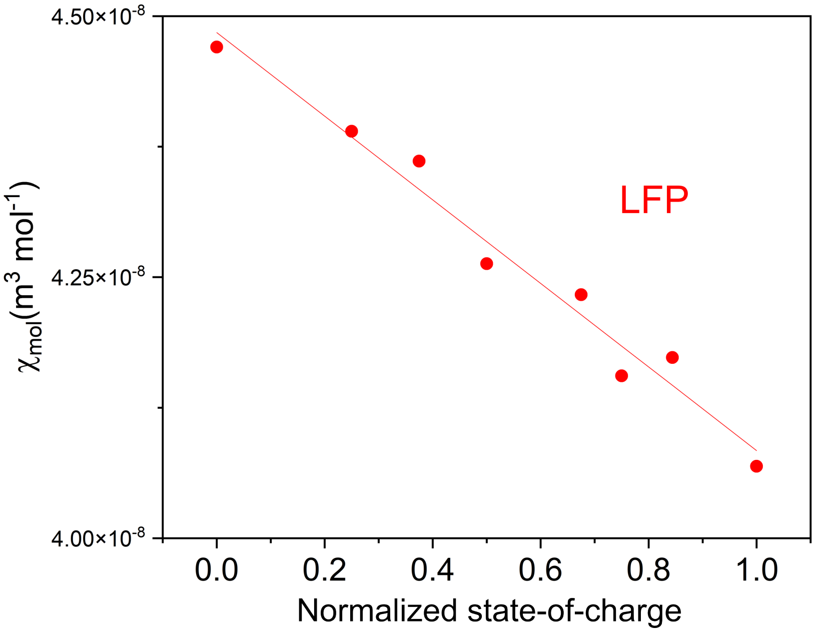

Figure S4: **Variation of molar magnetic susceptibility of LFP measured at different states of charge**. The LFP at different states of charge is a two-phase mixture of $LiFePO_4$ and $FePO_4$. Data from Ref. [4].

S2. Simulations - Solid-state Li-ion battery model

The numerical Li-ion battery model is used to simulate the charging process of a Li|$Li_7La_3Zr_2O_{12}$|Li_xCoO_2 solid-state battery (SSB) with the Li_xCoO_2 positive electrode particles containing various lithiation states and a lithium dendrite forming along the electrolyte grain boundaries. In the following, we describe the 3D COMSOL [5, 6] model in detail and provide references for the selected parameters.

S2.1. Geometrical setup

The configuration corresponds to in-plane SSBs, with ionic transport taking place in the plane of the electrolyte thin film with a thickness of $t = 5\ \mu$m. As shown in Fig. S5, the SSB has a width $w = 25\ \mu$m and its length $l = 50\ \mu$m is composed of the Li_xCoO_2|$Li_7La_3Zr_2O_{12}$ composite positive electrode $l_c = 25\ \mu$m, the solid-state electrolyte $l_{electrolyte} = 20\ \mu$m, and Li metal negative electrode $l_a = 5\ \mu$m. Particle sizes, particle shapes, and packing density are chosen to reflect X-ray tomography and scanning electron microscopy studies of $LiCoO_2$ [7] and LLZO [8].

As we are interested in investigating the magnetic signals of inhomogeneous battery operation, a lithium dendrite penetrates 15 μm into the solid-state electrolyte. It is buried 1 μm underneath the surface, its thickness is $t_{dendrite} = 2\ \mu$m, and it is growing along the grain boundaries of LLZO [9]. Its shape is further inspired by the branching morphology described in Ref. [10]. Other parameter choices and their respective references are given in Table S1.

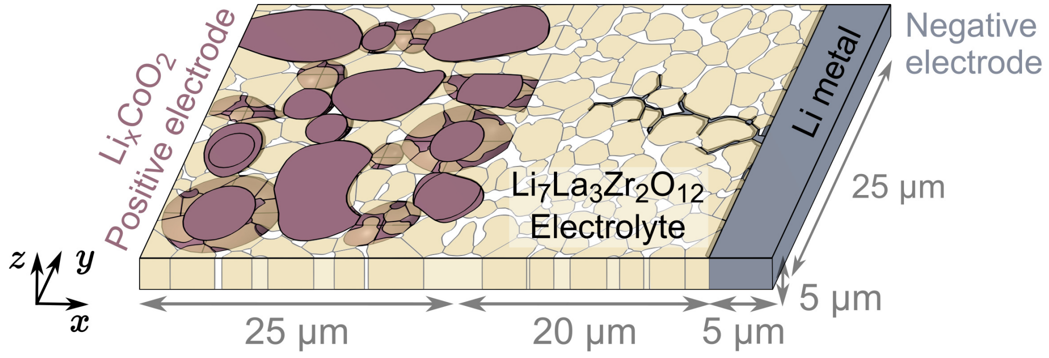

Figure S5: **Schematics of the Li-ion battery model in COMSOL.** The geometry is inspired by X-ray tomography and scanning electron microscopy studies.

S2.2. Input parameters and references

Table S1: **Parameters used in the finite element simulations of the numerical battery model.** Varying parameter values are marked by $f(\cdot)$.

Parameter	Value	Unit	Symbol
**Solid-state battery**			
Nominal 1C rate current density (Eq. 12)	0.94	$mA\cdot cm^{-2}$	$J_{1C,nominal}$
Nominal contact area	1.25×10^{-6}	cm^2	$A_{nominal}$
Positive collector area	1.23×10^{-7}	cm^2	$A_{collector^+}$
**LLZO**			
Electronic conductivity [11]	1×10^{-9}	$S\cdot cm^{-1}$	$\sigma_{e^-,LLZO}$
Ionic conductivity [12, 13]	5×10^{-4}	$S\cdot cm^{-1}$	σ_{LLZO}
Ionic conductivity (grain boundaries) [14, 15]	8.8×10^{-7}	$S\cdot cm^{-1}$	$\sigma_{bnd,LLZO}$
Volume magnetic susceptibility (measured here)	-7.16×10^{-8}	-	χ_{LLZO}
**Li_xCoO_2 (LCO)**			
Electronic conductivity [16]	$f(c_{Li^+})$	$S\cdot cm^{-1}$	σ_{LCO}
Constant electronic conductivity [17]	1.1×10^{-3}	$S\cdot cm^{-1}$	$\sigma_{LCO,const}$
Diffusivity [18]	$f(c_{Li^+})$	$cm^2\cdot s^{-1}$	D_{Li^+}
Constant diffusivity [17, 19]	5×10^{9}	$cm^2\cdot s^{-1}$	$D_{Li^+,const}$
Maximum concentration of Li-ions [17]	49937	$mol\cdot m^{-3}$	$c_{Li^+,max}$
Reference exchange current density [17, 20]	5.43×10^{-3}	$mA\cdot cm^{-2}$	$j_{0,ref,LCO}$
Negative electrode transfer coefficient [17]	0.5	-	α_a
Positive electrode transfer coefficient [17]	0.5	-	α_c
Double-layer capacitance (LLZO) [21]	$f(\phi_{LCO\|LLZO})$	$\mu F\cdot cm^{-2}$	$C_{LCO\|LLZO}$
Density [22]	4.79	$g\cdot cm^{-3}$	ρ_{LCO}
Volume	1.76×10^{-9}	cm^3	V_{LCO}
Volume magnetic susceptibility [2]	$f(c_{Li^+})$	-	χ_{LCO}
Theoretical capacity	274	$mAh\cdot g^{-1}$	
Reversible capacity [23]	140	$mAh\cdot g^{-1}$	Q^m_{LCO}
**Metallic lithium**			
Electronic conductivity [24]	1.03×10^{5}	$S\cdot cm^{-1}$	σ_{Li}
Reference exchange current density [17]	40	$mA\cdot cm^{-2}$	$j_{0,ref,Li}$
Double-layer capacitance (LLZO) [25]	10	$\mu F\cdot cm^{-2}$	$C_{LCO\|LLZO}$
Volume magnetic susceptibility [24]	1.09×10^{-6}	-	χ_{Li}

S2.3. Modeling approach

In this study, we use COMSOL [5], a finite element method framework, and its submodule "Lithium-ion Battery" to model the charging process of solid-state Li-ion batteries. The variables used in the equations below are defined in Table S1.

Simulations of LCO positive electrode. Inside Li_xCoO_2, the electronic current flow is governed by Ohm's law:

$$\mathbf{J}_{e^-,LCO} = -\sigma_{LCO}\nabla\phi_{LCO} \ . \tag{1}$$

The diffusion of Li-ions is modeled by Fick's law:

$$\mathbf{J}_{Li^+,LCO} = -D_{Li^+}\nabla\mathbf{c}_{Li^+} \ . \tag{2}$$

Simulations of LLZO electrolyte. As the electronic current in LLZO has a negligible contribution to the total current, we only model the ionic conductivity in the solid electrolyte and its grain boundaries:

$$\mathbf{J}_{Li^+,LLZO} = -(\sigma_{LLZO} + \sigma_{bnd,LLZO})\nabla\phi_{LLZO} \ . \tag{3}$$

Simulations of lithium negative electrode. Here, we only model the electronic current flow:

$$\mathbf{J}_{e^-,Li} = -\sigma_{Li}\nabla\phi_{Li} \ . \tag{4}$$

Other processes, e.g., the actual plating of lithium metal at the electrode surface, are not resolved dynamically for simplicity, but instead accounted for using a predefined lithium dendrite.

Electrode kinetics. Butler-Volmer kinetics are employed to model the redox reaction at the electrode interfaces to the electrolyte:

$$\mathbf{J}_{loc} = \mathbf{J}_0\left(\exp\left(\frac{\alpha_a F\eta}{RT}\right) - \exp\left(\frac{-\alpha_c F\eta}{RT}\right)\right) , \tag{5}$$

where $\mathbf{J}_{loc}$ is the local exchange current density, $\eta = \phi - \phi_{eq}$ is the local overpotential, $F = 96,485$ C·mol^{-1} is the Faraday constant, $R = 8.3145$ J·mol^{-1}·K^{-1} is the gas constant, and $T = 293.15$ K. The equilibrium potential of Li_xCoO_2, $\phi_{eq,LCO}$, is dependent on the lithium content x [26]. For metallic lithium, we set $\phi_{eq,Li} = 0$.

$\mathbf{J}_0$ can be derived as follows:

$$\mathbf{J}_{0,LCO} = j_{0,ref,LCO}\left(\frac{2\mathbf{c}_{Li^+}}{c_{Li^+,max}}\right)^{\alpha_c}\left(\frac{2c_{Li^+,max} - 2\mathbf{c}_{Li^+}}{c_{Li^+,max}}\right)^{\alpha_a} , \tag{6}$$

$$\mathbf{J}_{0,Li} = j_{0,ref,Li} \ , \tag{7}$$

where c_{Li^+} is the Li-ion concentration measured on the surface of the active particles. The initial concentration of Li-ions is $c_{\mathrm{Li}^+,\mathrm{init}} = 48938$ mol·m^{-3}. This evaluates to an initial state-of-charge $\mathrm{x}_{\mathrm{init}} = 0.98$. To prevent irreversible delithiation, we set the minimum average lithium content of the $\mathrm{Li_xCoO_2}$ electrode to $\mathrm{x}_{\mathrm{min}} = 0.47$. The available charge $Q_{\mathrm{LCO}} = 4.32 \times 10^{-6}$ C $= 1.20 \times 10^{-6}$ mAh can then be calculated as follows:

$$Q_{\mathrm{LCO}} = (\mathrm{x}_{\mathrm{init}} - \mathrm{x}_{\mathrm{min}}) \cdot c_{\mathrm{Li}^+,\mathrm{max}} \cdot F \cdot V_{\mathrm{LCO}} \ . \tag{8}$$

The capacitance between the electrode|electrolyte interfaces, i.e., a space-charge layer of about a nanometer in thickness [21], is described by $C_{\mathrm{LCO|LLZO}}$ and $C_{\mathrm{LCO|LLZO}}$, respectively.

Ørsted field calculation. Here, we use COMSOL's submodule "Magnetic Fields, Currents Only". The finite element method solves Ampère's law:

$$\nabla \times \mathbf{B}_{\mathrm{Ørsted}} = \mu_0 \mathbf{J}_{\mathrm{SSB}} \ , \tag{9}$$

where $\mathbf{J}_{\mathrm{SSB}} = \mathbf{J}_{\mathrm{e}^-,\mathrm{LCO}} + \mathbf{J}_{\mathrm{Li}^+,\mathrm{LLZO}} + \mathbf{J}_{\mathrm{e}^-,\mathrm{Li}}$.

Magnetic field from applied magnetic flux density. Here, we use COMSOL's submodule "Magnetic Fields, No Currents". We apply an external magnetic field strength $B_{\mathrm{ext}} = 100$ mT, which magnetizes the battery components according to their volume magnetic susceptibility $\mu_0 \mathbf{M} = \chi_{\mathrm{v}} B_{\mathrm{ext}}$. The resulting fields are then summed up:

$$\mathbf{B} = \mu_0 (\mathbf{H} + \mathbf{M}_{\mathrm{LCO}} + \mathbf{M}_{\mathrm{LLZO}} + \mathbf{M}_{\mathrm{Li}}) \ , \tag{10}$$

where $\mathbf{H} = -\nabla \phi_m$ is the demagnetizing field strength calculated from the magnetic scalar potential ϕ_m. This can be derived from the known magnetization [27].

1C rate calculation. When only taking into account the actual contact area to $\mathrm{Li_xCoO_2}$ in the positive x-direction and skipping other poor conducting regions such as LLZO, the positive current collector has an area $A_{\mathrm{collector}^+} = 1.23 \times 10^{-7}$ cm^2. With these values, the 1C rate of the model is calculated as follows:

$$J_{\mathrm{1C}} = \frac{V_{\mathrm{LCO}} \cdot \rho_{\mathrm{LCO}} \cdot Q^m_{\mathrm{LCO}}}{1\mathrm{h} \cdot A_{\mathrm{collector}^+}} = 9.59 \qquad [\mathrm{mA{\cdot}cm^{-2}}] \ . \tag{11}$$

The nominal current density, which is normally reported, is the current flowing through the full area, i.e., summing active and inactive regions, on the positive current collector side $A_{\mathrm{nominal}} = t \cdot w = 1.25 \times 10^{-6}$ cm^2:

$$J_{\mathrm{1C,nominal}} = J_{\mathrm{1C}} \cdot \frac{A_{\mathrm{collector}^+}}{A_{\mathrm{nominal}}} = 0.94 \qquad [\mathrm{mA{\cdot}cm^{-2}}] \ . \tag{12}$$

S2.4. Procedure to obtain state-of-charge of Fig. 4 in the main text

To get to the state-of-charge of the solid-state Li-ion battery presented in Fig. 4 in the main text, the battery is charged for 4 min with J_{5C} starting from $x_{init} = 0.98$. This results in a lithium concentration gradient across the positive electrode spanning from $Li_{0.93}CoO_2$ to $Li_{0.53}CoO_2$.

In Fig. 4a in the main text, the current density plot is derived from running the simulation for 0.6 s with J_{25C}. The resulting $\mathbf{B}_{Ørsted}$ is simulated 50 nm above the battery surface in the xy-plane. Similarly, the paramagnetic field resulting from the induced magnetization in an external magnetic flux density $B_{ext} = 100$ mT applied in the positive z-direction is measured 50 nm above the surface.

The curves, presented on the left in Fig. 4b in the main text, show $\mathbf{B}_z$, i.e., the z-component of the paramagnetic signal, centered 50 nm above the battery surface of two selected positive electrode particles and the lithium metal negative electrode, respectively. The two positive electrode particles are shown in Fig. S6. The paramagnetic signal strength is linearly extrapolated for external magnetic fields ranging from 0.1 mT to 1 T, which is well-justified by the visible linearity of the magnetization curves of Li_xCoO_2 samples shown in Fig. S2.

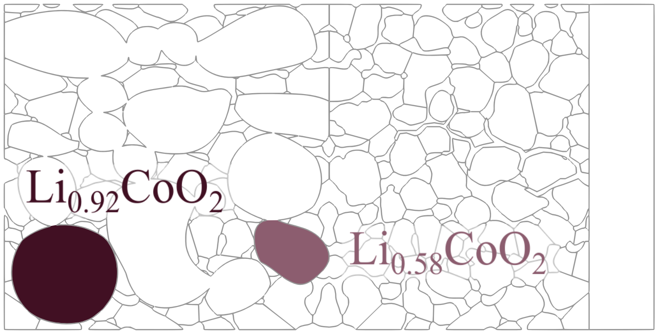

Figure S6: **Selected positive electrode particles for evaluating the paramagnetic field.** These particles are used for the paramagnetic field strength plot presented on the left in Fig. 4b in the main text.

In the line plot for the signal strength from the Ørsted field, which is shown on the right in Fig. 4b in the main text, we obtain data points for different nominal current densities by running short simulations (< 1 s) using an average state-of-charge of $Li_{0.81}CoO_2$ as a starting point. This starting point is obtained by charging for 4 min with J_{5C} to induce heterogeneous lithium composition in $LiCoO_2$, as shown in Fig. S8c. The duration of the simulations used to calculate the Ørsted field is chosen to be sufficiently short so that its effect on the state-of-charge of the battery is negligible. The Ørsted field is evaluated along vertical cuts through the battery as shown in Fig. S7, with the maximum value along these lines defining the magnetic signal strength used in Fig. 4b in the main text.

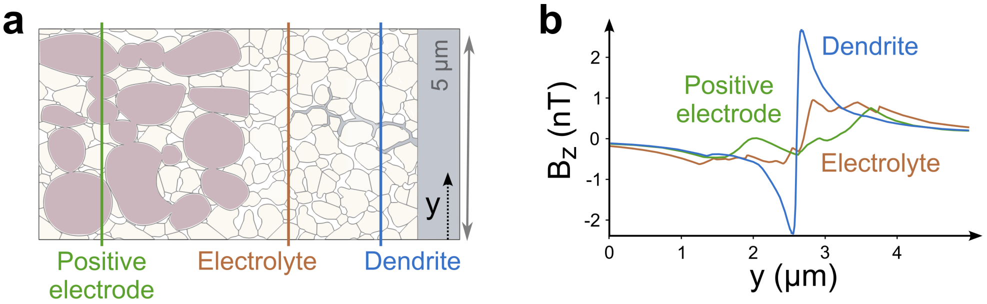

Figure S7: **Evaluation of the Ørsted magnetic field.** (a) Schematics and respective line placement in y-direction, where the Ørsted field is measured. (b) Example stray field generated 50 nm above the battery surface by J_{25C}. The maximum values of these lines are presented in Fig. 4b in the main text for a wide range of applied current densities.

S2.5. Lithium concentration after charging with C/10, 1C, and 5C rate

In Fig. S8, we show the lithium content in positive electrode particles for different C-rates (a-c) along with the magnetic field 50 nm above the battery surface produced by the paramagnetic components in an external field of 100 mT (d-f), the electronic conductivity dependent on the lithiation state of the individual particles (g-i), the resulting current density when a voltage of 1 V is applied across the positive electrode (j-l), and the Ørsted field produced by that current 50 nm above the positive electrode surface (m-o). The respective charging durations are chosen to obtain a state-of-charge with approximately a third of the reversible amount of lithium transferred in all three cases. Hence, the average state-of-charge is expected to be $Li_{0.81}CoO_2$. It can be seen that the lithium content is distributed evenly with $J_{C/10,\mathrm{nominal}}$, with the lithium content inside the $LiCoO_2$ particles spanning from x = 0.80 to x = 0.79 (Fig. S8a). For $J_{1C,\mathrm{nominal}}$ in Fig. S8b, the lithiation states are evaluated to range from x = 0.86 to x = 0.69. Whereas for $J_{5C,\mathrm{nominal}}$, the more heterogeneous redox reactivity has driven the $LiCoO_2$ particles to lithiation states spanning from x = 0.93 to x = 0.53, and a gradient in lithium content at 5C rate becomes visible between individual particles (Fig. S8c). This behavior is directly reflected in the paramagnetic field measured 50 nm above the positive electrode surface, see Fig. S8d-f, which is due to the magnetic susceptibility dependence of Li_xCoO_2 on the lithiation state, as the magnetization curves show in Fig. S2.

More details on how the voltage of 1 V is applied across the positive electrode are given in Fig. S9. After charging with a rate of 5C for 4 min, we place the positive electrode between two additional electrodes and apply a voltage of 1 V across them. As σ_{LCO} depends on the lithiation state of the individual particles, a heterogeneous current density map is obtained inside the positive electrode, resulting in a feature-rich Ørsted field 50 nm above the surface. In contrast to Fig. 4 in the main text, these magnetic field values exceed the continuous-wave optically detected magnetic resonance (CW-ODMR) detection limit, which opens up the possibility to visualize the charge flow through reconstruction.

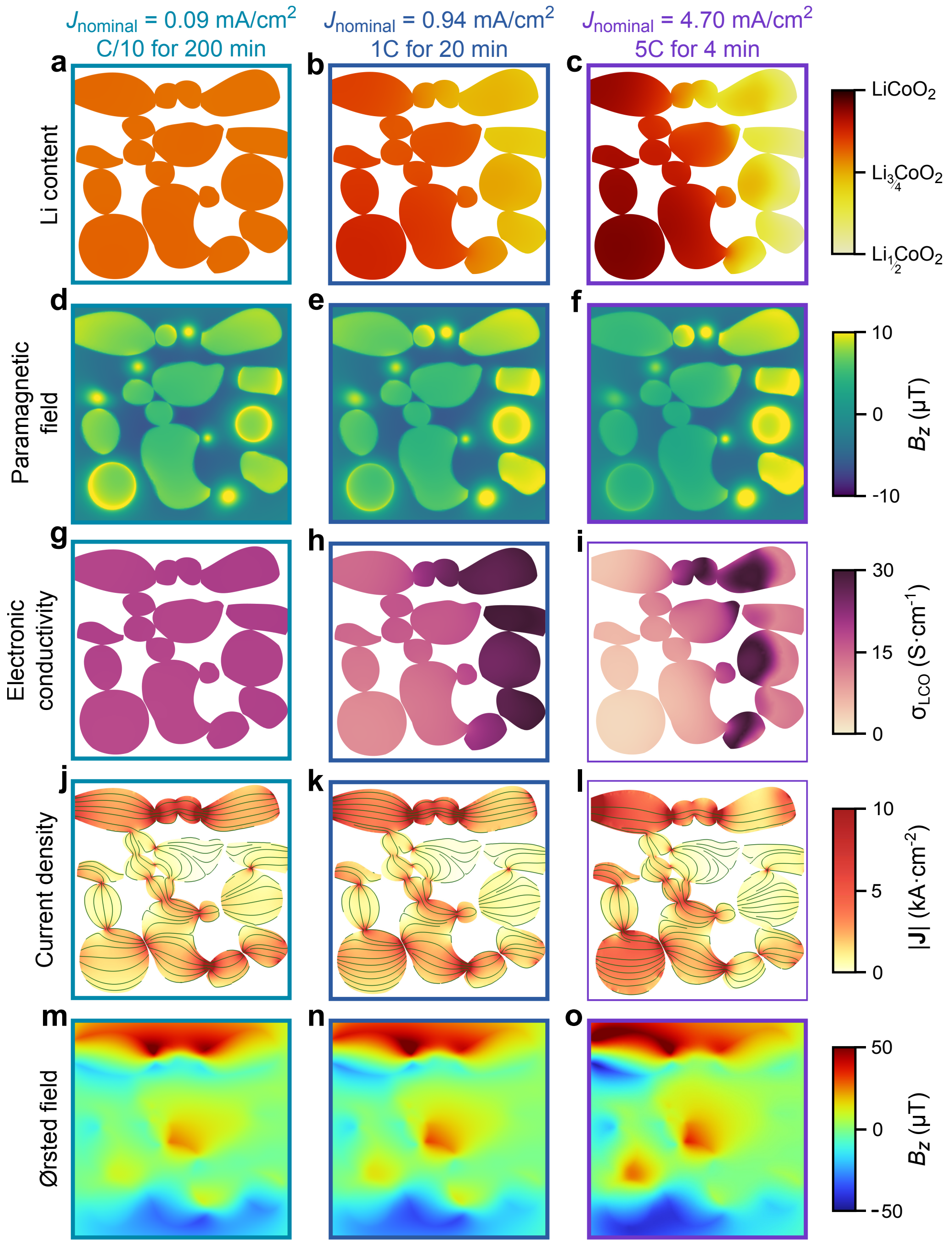

Figure S8: **Positive electrode characteristics after charging the battery with different C-rates for approximately a third of the reversible amount of lithium $Q^m_{\mathbf{LCO}}$.** The following presented 2D plots, (a-c) lithium content, (g-i) electronic conductivity, and (j-l) current density obtained with an applied voltage of 1 V across the positive electrode, are cross-sections through the positive electrode parallel to the xy-plane at $z = 3.5\ \mu$m. The magnetic field maps, i.e., (d-f) paramagnetic field induced by an external field of 100 mT in the positive z-direction, and (m-o) Ørsted field, are measured 50 nm above the positive electrode surface. Due to the variation in lithiation states when charging at different C-rates, the positive electrode particles feature distinguishable magnetic and electronic characteristics.

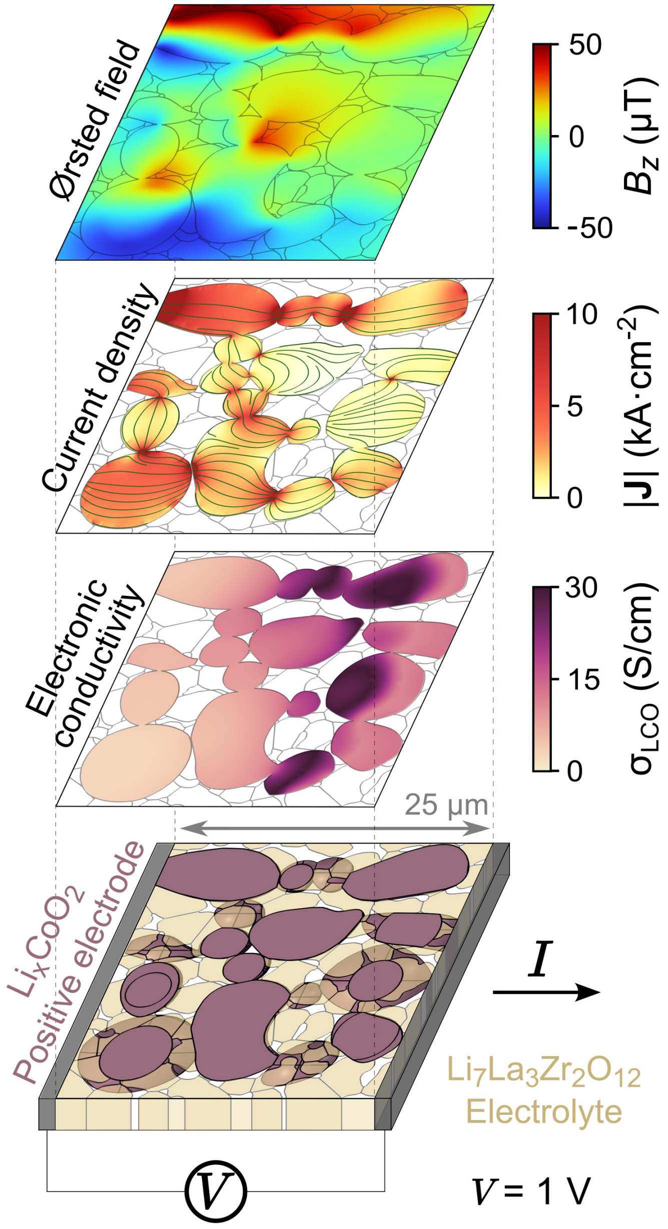

Figure S9: **Finite element simulations of applying 1 V across an Li_xCoO_2 positive electrode, which had been charged at a rate of 5C for 4 min.** Above the setup shown in the bottom, the electronic conductivities of Li_xCoO_2 particles of various lithiation states, the simulated current density at a cross-section parallel to the xy-plane at $z = 3.5$ μm, and the resulting Ørsted field 50 nm above the positive electrode surface.

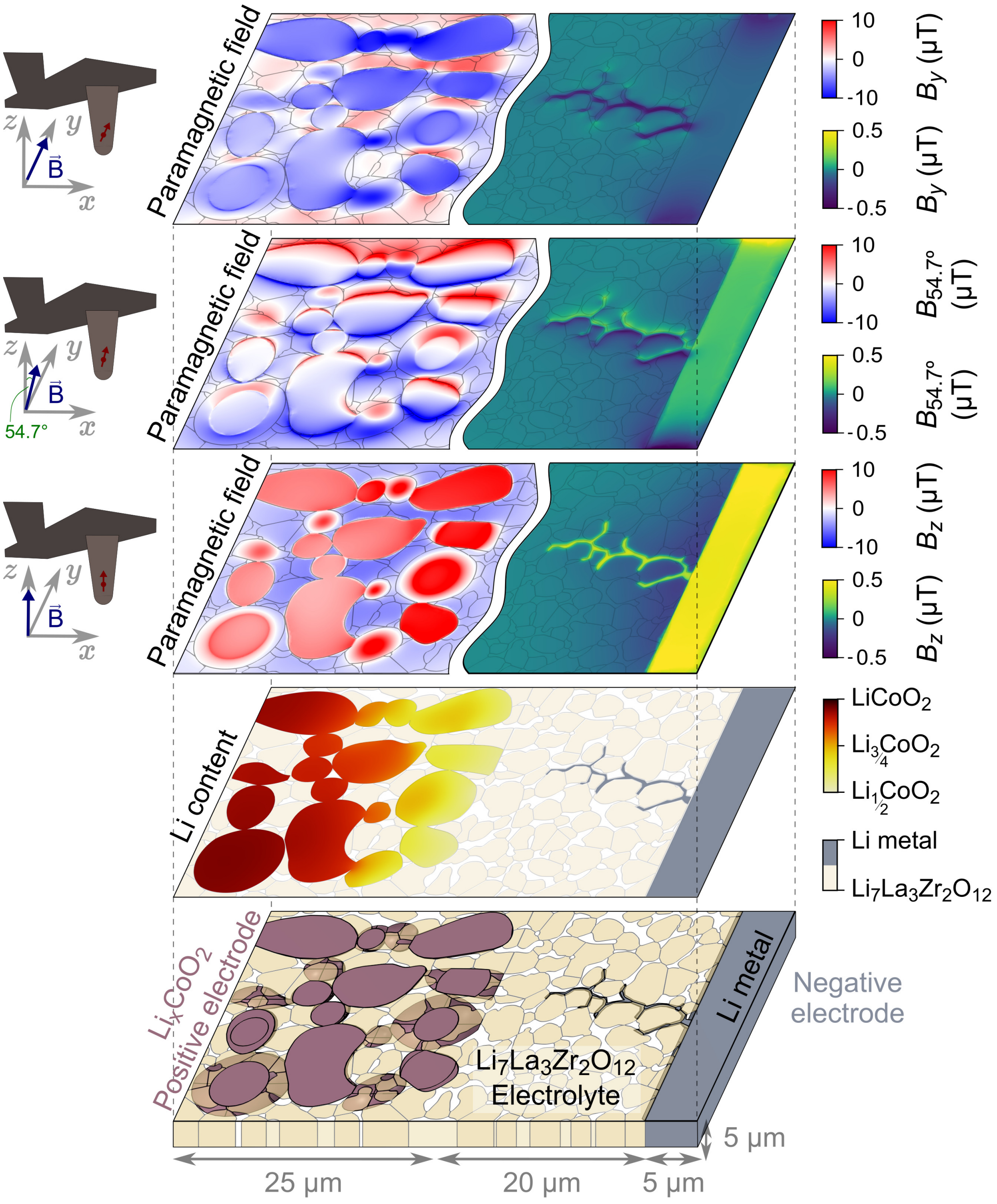

Figure S10: **Finite element simulations of a $Li|Li_7La_3Zr_2O_{12}|Li_xCoO_2$ solid-state battery with the Li_xCoO_2 positive electrode particles containing various lithiation states and a lithium metal dendrite forming in the $Li_7La_3Zr_2O_{12}$ electrolyte grain boundaries.** In the lower panels, the schematics of the battery and the lithium content of the Li_xCoO_2 positive electrode after charging with 5C rate for 4 min, along with the lithium metal locations. In the center panel, the magnetic field produced by the paramagnetic components in an external field of 100 mT aligned with the NV axis orientation in the positive z-direction is shown. In the upper two panels, we show the paramagnetic field in an external field of 100 mT aligned with NV axis orientations of $54,7°$ with respect to the z-axis and in the positive y-direction, respectively.

S2.6. Paramagnetic field from different NV axis orientations

In Fig. 4a in the main text, we visualize the magnetic field produced by the battery's paramagnetic components in an external field of 100 mT. Here, both the external magnetic field axis and, accordingly, the paramagnetic moments are pointing in the positive z-direction and are aligned with the sensor's nitrogen vacancy (NV) axis orientation. This is more explicitly shown in the center panel of Fig. S10, where B_z is depicted 50 nm above the battery surface. Additionally, two other NV axis orientations are available in commercial systems [28], which are depicted in the upper panels in Fig. S10 with $B_{54.7^\circ}$ and B_y. Each NV axis orientation results in a distinctive paramagnetic field pattern.

S3. Characteristics of magnetic microscopy techniques

In the following, we have listed spatial resolution, typical field-of-view specifications, and other properties for various imaging techniques. Hereby, the following abbreviations are used:

- Scanning Electrochemical Microscopy (SECM)
- Scanning Ion-Conductance Microscopy (SICM)
- Kelvin Probe Force Microscopy (KPFM)
- Conductive Atomic Force Microscopy (C-AFM)
- Electrochemical Strain Microscopy (ESM)
- Tip-Enhanced Raman Spectroscopy (TERS)
- Scanning Transmission X-Ray Microscopy (STXM)
- High-Resolution Transmission Electron Microscopy (HR-TEM)
- Scanning Electron Microscopy (SEM)
- Superconducting Quantum Interference Device (SQUID)
- Nitrogen-Vacancy (NV)

S3.1. Overview characterization methods

Table S2: **Overview of selected imaging techniques used for characterizing batteries.**
($*$) *Limited to magnetic stray field*; ($\dagger$) *Limited to surface topography*; ($\ddagger$) *Except nanoscale NMR*; ($\diamond$) *Interaction with sample, but is non-destructive*; ($\ominus$) *Beam influence possible*; ($\otimes$) *Beam damage*; ($\odot$) *May be influenced by large magnetic fields*; ($\square$) *Needs validation.*

	**Technique**	**Spatial resolution [nm]**	**Field of view [μm]**	**Application to ”buried” samples**	**Imaging currents**	**Chemical details**	**Structural details**	**Non-invasive-ness**
**Scanning probes**	SECM & SICM [29, 30]	100–1,000	100	No	Yes	Yes	Yes†	No$^{\diamond}$
	KPFM & C-AFM [31–33]	1–10	200	No	Yes	No	Yes†	No$^{\diamond}$
	ESM [34, 35]	1–10	100	No	Yes	No	Yes†	No$^{\diamond}$
**Photon**	TERS [36–39]	< 1	1	No	No	Yes	Yes	Yes
	Raman microscopy [40]	1,000	10,000	Yes	No	Yes	Yes	Yes
	X-ray nanotomography [41]	10	100	Yes	No	Yes	Yes	Yes$^{\ominus}$
	STXM [42, 43]	20	1	Yes	No	Yes	Yes	Yes$^{\ominus}$
**Electron**	HR-TEM [31, 44–46]	0.1	0.01	No	No	Yes	Yes	No$^{\otimes}$
	SEM [31, 47, 48]	1–10	1	No	No	Yes	Yes	No$^{\otimes}$
**Magnetic**	Magnetic resonance [49]	10,000	10,000	Yes*	No	Yes	Yes	Yes$^{\odot}$
	Atomic Magnetometers [50–53]	1,000,000	10,000	Yes*	Yes	Yes	No	Yes
	Scanning SQUID [54–56]	200–500	100	Yes*	Yes	Yes	Yes†	Yes
	Scanning NV [28, 57]	10	20	Yes*	Yes	Yes	Yes†	Yes$^{\square}$
	Widefield NV [58]	1,000	1,000	Yes*	Yes	Yes	No‡	Yes$^{\square}$

S3.2. Benchmark of magnetic imaging techniques

Table S3: **Comparison between commercial magnetic imaging techniques.** We note that at the time of writing, the production of the commercial scanning SQUID setup from FormFactor [54] has been discontinued.

	**Spatial resolution**	**Field of view**	**Sensitivity**	**Frequency resolution**	**Operating temperature**
**Scanning SQUID** [54–56]	200-500 nm	100 μm	1 nT $\cdot$ Hz$^{-0.5}$	DC to MHz	Cryogenic 3-20 K
**Atomic magnetometers** [50]	1 mm	1 cm	10 fT $\cdot$ Hz$^{-0.5}$	DC to kHz	Ambient conditions
**Scanning NV** [28, 57]	10 nm	20 μm	1 μT $\cdot$ Hz$^{-0.5}$	DC to GHz	mK to $\gg$ 300 K
**Widefield NV** [58]	1 μm	1 mm	1 μT $\cdot$ Hz$^{-0.5}$	DC to GHz	mK to $\gg$ 300 K

Supplementary references

[1] G. Klinser, S. Topolovec, H. Kren, S. Koller, H. Krenn, R. Würschum, Charging of lithium cobalt oxide battery cathodes studied by means of magnetometry, Solid State Ionics 293 (2016) 64–71.

[2] K. Mukai, Y. Ikedo, H. Nozaki, J. Sugiyama, K. Nishiyama, D. Andreica, A. Amato, P. Russo, E. Ansaldo, J. Brewer, et al., Magnetic phase diagram of layered cobalt dioxide li x coo 2, Physical review letters 99 (2007) 087601.

[3] A. J. Ilott, M. Mohammadi, C. M. Schauerman, M. J. Ganter, A. Jerschow, Rechargeable lithium-ion cell state of charge and defect detection by in-situ inside-out magnetic resonance imaging, Nature Communications 9 (2018).

[4] T. Gallien, H. Krenn, R. Fischer, S. Lauterbach, B. Schweighofer, H. Wegleiter, Magnetism versus lifepo4 battery's state of charge: A feasibility study for magnetic-based charge monitoring, IEEE Transactions on Instrumentation and Measurement 64 (2015) 2959–2964.

[5] S. COMSOL AB, Stockholm, Comsol multiphysics® v. 6.2, 2023. URL: www.comsol.com.

[6] S. Pollok, M. Khoshkalam, F. Ghaffari-Tabrizi, D. V. Christensen, Comsol solid-state li-ion battery model, 2025. URL: https://doi.org/10.11583/DTU.29485181. doi:10.11583/DTU.29485181.

[7] Z. Liu, J. S. Cronin, K. Yu-chen, J. R. Wilson, K. J. Yakal-Kremski, J. Wang, K. T. Faber, S. A. Barnett, Three-dimensional morphological measurements of licoo2 and licoo2/li (ni1/3mn1/3co1/3) o2 lithium-ion battery cathodes, Journal of Power Sources 227 (2013) 267–274.

[8] M. Abreu-Sepúlveda, D. E. Williams, A. Huq, C. Dhital, Y. Li, M. P. Paranthaman, K. Zaghib, A. Manivannan, Synthesis and characterization of substituted garnet and perovskite-based lithium-ion conducting solid electrolytes, Ionics 22 (2016) 317–325.

[9] E. J. Cheng, A. Sharafi, J. Sakamoto, Intergranular li metal propagation through polycrystalline li6. 25al0. 25la3zr2o12 ceramic electrolyte, Electrochimica Acta 223 (2017) 85–91.

[10] E. Kazyak, R. Garcia-Mendez, W. S. LePage, A. Sharafi, A. L. Davis, A. J. Sanchez, K.-H. Chen, C. Haslam, J. Sakamoto, N. P. Dasgupta, Li penetration in ceramic solid electrolytes: operando microscopy analysis of morphology, propagation, and reversibility, Matter 2 (2020) 1025–1048.

[11] T. Thompson, S. Yu, L. Williams, R. D. Schmidt, R. Garcia-Mendez, J. Wolfenstine, J. L. Allen, E. Kioupakis, D. J. Siegel, J. Sakamoto, Electrochemical window of the li-ion solid electrolyte li7la3zr2o12, ACS Energy Letters 2 (2017) 462–468.

[12] T. Krauskopf, B. Mogwitz, H. Hartmann, D. K. Singh, W. G. Zeier, J. Janek, The fast charge transfer kinetics of the lithium metal anode on the garnet-type solid electrolyte li6. 25al0. 25la3zr2o12, Advanced Energy Materials 10 (2020) 2000945.

[13] R. Murugan, V. Thangadurai, W. Weppner, et al., Fast lithium ion conduction in garnet-type li7la3zr2o12, ANGEWANDTE CHEMIE-INTERNATIONAL EDITION IN ENGLISH- 46 (2007) 7778.

[14] H. Xie, C. Li, W. H. Kan, M. Avdeev, C. Zhu, Z. Zhao, X. Chu, D. Mu, F. Wu, Consolidating the grain boundary of the garnet electrolyte llzto with li 3 bo 3 for high-performance lini 0.8 co 0.1 mn 0.1 o 2/lifepo 4 hybrid solid batteries, Journal of Materials Chemistry A 7 (2019) 20633–20639.

[15] W. E. Tenhaeff, Y. Wang, A. P. Sokolov, J. Wolfenstine, J. Sakamoto, N. J. Dudney, E. Rangasamy, Resolving the grain boundary and lattice impedance of hot-pressed li7la3zr2o12 garnet electrolytes, ChemElectroChem 1 (2013).

[16] M. Ménétrier, I. Saadoune, S. Levasseur, C. Delmas, The insulator-metal transition upon lithium deintercalation from licoo2: electronic properties and 7li nmr study, Journal of Materials Chemistry 9 (1999) 1135–1140.

[17] K. V. Kravchyk, D. T. Karabay, M. V. Kovalenko, On the feasibility of all-solid-state batteries with llzo as a single electrolyte, Scientific Reports 12 (2022) 1177.

[18] J. Barker, R. Pynenburg, R. Koksbang, M. Saidi, An electrochemical investigation into the lithium insertion properties of lixcoo2, Electrochimica acta 41 (1996) 2481–2488.

[19] Y.-M. Choi, S.-I. Pyun, J.-S. Bae, S.-I. Moon, Effects of lithium content on the electrochemical lithium intercalation reaction into linio2 and licoo2 electrodes, Journal of power sources 56 (1995) 25–30.

[20] J. Sastre, X. Chen, A. Aribia, A. N. Tiwari, Y. E. Romanyuk, Fast charge transfer across the li7la3zr2o12 solid electrolyte/licoo2 cathode interface enabled by an interphase-engineered all-thin-film architecture, ACS applied materials & interfaces 12 (2020) 36196–36207.

[21] N. J. de Klerk, M. Wagemaker, Space-charge layers in all-solid-state batteries; important or negligible?, ACS applied energy materials 1 (2018) 5609–5618.

[22] E. J. Cheng, N. J. Taylor, J. Wolfenstine, J. Sakamoto, Elastic properties of lithium cobalt oxide (licoo2), Journal of Asian Ceramic Societies 5 (2017) 113–117.

[23] H. Xia, L. Lu, Y. S. Meng, G. Ceder, Phase transitions and high-voltage electrochemical behavior of licoo2 thin films grown by pulsed laser deposition, Journal of The Electrochemical Society 154 (2007) A337.

[24] R. C. Weast, Crc handbook of chemistry and physics. (1986).

[25] A. Sharafi, H. M. Meyer, J. Nanda, J. Wolfenstine, J. Sakamoto, Characterizing the li–li7la3zr2o12 interface stability and kinetics as a function of temperature and current density, Journal of Power Sources 302 (2016) 135–139.

[26] M. Tang, P. Albertus, J. Newman, Two-dimensional modeling of lithium deposition during cell charging, Journal of The Electrochemical Society 156 (2009) A390.

[27] F. Slanovc, M. Ortner, M. Moridi, C. Abert, D. Suess, Full analytical solution for the magnetic field of uniformly magnetized cylinder tiles, Journal of Magnetism and Magnetic Materials 559 (2022) 169482.

[28] QZabre, Qzabre, 2018. URL: https://qzabre.com/en.

[29] C. G. Zoski, advances in scanning electrochemical microscopy (secm), Journal of The Electrochemical Society 163 (2015) H3088.

[30] C. Zhu, K. Huang, N. P. Siepser, L. A. Baker, Scanning Ion Conductance Microscopy, Chemical Reviews 121 (2021) 11726–11768. URL: https://doi.org/10.1021/acs.chemrev.0c00962. doi:10.1021/acs.chemrev.0c00962, publisher: American Chemical Society.

[31] M. B. Dixit, J.-S. Park, P. Kenesei, J. Almer, K. B. Hatzell, Status and prospect of in situ and operando characterization of solid-state batteries, Energy & Environmental Science 14 (2021) 4672–4711.

[32] K. Bian, C. Gerber, A. J. Heinrich, D. J. Müller, S. Scheuring, Y. Jiang, Scanning probe microscopy, Nature Reviews Methods Primers 1 (2021) 36.

[33] Z. Zhang, S. Said, K. Smith, R. Jervis, C. A. Howard, P. R. Shearing, D. J. Brett, T. S. Miller, Characterizing batteries by in situ electrochemical atomic force microscopy: a critical review, Advanced Energy Materials 11 (2021) 2101518.

[34] R. Giridharagopal, L. Flagg, J. Harrison, M. Ziffer, J. Onorato, C. Luscombe, D. Ginger, Electrochemical strain microscopy probes morphology-induced variations in ion uptake and performance in organic electrochemical transistors, Nature materials 16 (2017) 737–742.

[35] S. Jesse, A. Kumar, T. M. Arruda, Y. Kim, S. V. Kalinin, F. Ciucci, Electrochemical strain microscopy: Probing ionic and electrochemical phenomena in solids at the nanometer level, MRS bulletin 37 (2012) 651–658.

[36] F. Latorre, S. Kupfer, T. Bocklitz, D. Kinzel, S. Trautmann, S. Gräfe, V. Deckert, Spatial resolution of tip-enhanced raman spectroscopy–dft assessment of the chemical effect, Nanoscale 8 (2016) 10229–10239.

[37] M. D. Sonntag, E. A. Pozzi, N. Jiang, M. C. Hersam, R. P. Van Duyne, Recent advances in tip-enhanced raman spectroscopy, The journal of physical chemistry letters 5 (2014) 3125–3130.

[38] Y. Cao, M. Sun, Tip-enhanced raman spectroscopy, Reviews in Physics 8 (2022) 100067.

[39] T. Deckert-Gaudig, A. Taguchi, S. Kawata, V. Deckert, Tip-enhanced raman spectroscopy–from early developments to recent advances, Chemical Society Reviews 46 (2017) 4077–4110.

[40] E. Smith, G. Dent, Modern Raman spectroscopy: a practical approach, John Wiley & Sons, 2019.

[41] J. Scharf, M. Chouchane, D. P. Finegan, B. Lu, C. Redquest, M.-c. Kim, W. Yao, A. A. Franco, D. Gostovic, Z. Liu, et al., Bridging nano-and microscale x-ray tomography for battery research by leveraging artificial intelligence, Nature Nanotechnology 17 (2022) 446–459.

[42] J. Kim, D. Lee, C. Nam, J. Chung, B. Koo, N. Kim, J. Lim, Energy material analysis via in-situ/operando scanning transmission x-ray microscopy: A review, Journal of Electron Spectroscopy and Related Phenomena 266 (2023) 147337.

[43] Y. Takeichi, Scanning transmission x-ray microscopy, Compendium of Surface and Interface Analysis (2018) 593–597.

[44] D. Schäfer, K. Hankins, M. Allion, U. Krewer, F. Karcher, L. Derr, R. Schuster, J. Maibach, S. Mück, D. Kramer, et al., Multiscale investigation of sodium-ion battery anodes: Analytical techniques and applications, Advanced Energy Materials 14 (2024) 2302830.

[45] W. Li, D. M. Lutz, L. Wang, K. J. Takeuchi, A. C. Marschilok, E. S. Takeuchi, Peering into batteries: electrochemical insight through in situ and operando methods over multiple length scales, Joule 5 (2021) 77–88.

[46] C. B. Carter, D. B. Williams, Transmission electron microscopy: Diffraction, imaging, and spectrometry, Springer, 2016.

[47] A. Khursheed, Scanning electron microscopy, Encyclopedia of Condensed Matter Physics (Second Edition) (2022).

[48] J. I. Goldstein, D. E. Newbury, J. R. Michael, N. W. Ritchie, J. H. J. Scott, D. C. Joy, Scanning electron microscopy and X-ray microanalysis, springer, 2017.

[49] H. Lin, Y. Jin, M. Tao, Y. Zhou, P. Shan, D. Zhao, Y. Yang, Magnetic resonance imaging techniques for lithium-ion batteries: Principles and applications: Dedicated to the special issue "magnetic resonance of electrochemical energy storage materials", Magnetic Resonance Letters (2024) 200113.

[50] QuSpin, Qzfm gen-3, 2021. URL: https://quspin.com/products-qzfm/.

[51] X. Bai, K. Wen, D. Peng, S. Liu, L. Luo, Atomic magnetometers and their application in industry, Frontiers in Physics 11 (2023). URL: https://www.frontiersin.org/journals/physics/articles/10.3389/fphy.2023.1212368/full. doi:10.3389/fphy.2023.1212368, publisher: Frontiers.

[52] Y. Hu, G. Z. Iwata, M. Mohammadi, E. V. Silletta, A. Wickenbrock, J. W. Blanchard, D. Budker, A. Jerschow, Sensitive magnetometry reveals inhomogeneities in charge storage and weak transient internal currents in Li-ion cells, Proceedings of the National Academy of Sciences 117 (2020) 10667–10672. URL: https://www.pnas.org/doi/full/10.1073/pnas.1917172117. doi:10.1073/pnas.1917172117, publisher: Proceedings of the National Academy of Sciences.

[53] M. V. Romalis, H. B. Dang, Atomic magnetometers for materials characterization, Materials Today 14 (2011) 258–262. URL: https://www.sciencedirect.com/science/article/pii/S1369702111701407. doi:10.1016/S1369-7021(11)70140-7.

[54] FormFactor, Scanning squid microscope – accelerating quantum computing development, 2021. URL: https://www.formfactor.com/blog/2021/scanning-squid-microscope-accelerating-quantum-computing-development/.

[55] E. Persky, I. Sochnikov, B. Kalisky, Studying quantum materials with scanning squid microscopy, Annual Review of Condensed Matter Physics 13 (2022) 385–405.

[56] E. Marchiori, L. Ceccarelli, N. Rossi, L. Lorenzelli, C. L. Degen, M. Poggio, Nanoscale magnetic field imaging for 2d materials, Nature Reviews Physics 4 (2022) 49–60.

[57] Qnami, Proteusq - quantum microscope system, 2020. URL: https://qnami.ch/portfolio/proteusq/.

[58] QDM.IO, Our product, 2023. URL: https://qdm.io/our-product.
